# Challenges and Perspectives of Standard Therapy and Drug Development in High-Grade Gliomas

**DOI:** 10.3390/molecules26041169

**Published:** 2021-02-22

**Authors:** Shalini Sundramurthi Chelliah, Ervin Ashley Lourdes Paul, Muhamad Noor Alfarizal Kamarudin, Ishwar Parhar

**Affiliations:** 1 Brain Research Institute Monash Sunway, Jeffrey Cheah School of Medicine and Health Science, Monash University Malaysia, Bandar Sunway 47500, Malaysia; ssun95@student.monash.edu (S.S.C.); ealou1@student.monash.edu (E.A.L.P.); muhamadnoor.alfarizal@monash.edu (M.N.A.K.); 2 School of Science, Monash University Malaysia, Bandar Sunway 47500, Malaysia

**Keywords:** high-grade glioma, glioblastoma, anaplastic astrocytoma, anaplastic oligodendroglioma, oligodendroglioma, chemotherapy, radiotherapy, immunotherapy, phytochemicals, nanoparticles

## Abstract

Despite their low incidence rate globally, high-grade gliomas (HGG) remain a fatal primary brain tumor. The recommended therapy often is incapable of resecting the tumor entirely and exclusively targeting the tumor leads to tumor recurrence and dismal prognosis. Additionally, many HGG patients are not well suited for standard therapy and instead, subjected to a palliative approach. HGG tumors are highly infiltrative and the complex tumor microenvironment as well as high tumor heterogeneity often poses the main challenges towards the standard treatment. Therefore, a one-fit-approach may not be suitable for HGG management. Thus, a multimodal approach of standard therapy with immunotherapy, nanomedicine, repurposing of older drugs, use of phytochemicals, and precision medicine may be more advantageous than a single treatment model. This multimodal approach considers the environmental and genetic factors which could affect the patient’s response to therapy, thus improving their outcome. This review discusses the current views and advances in potential HGG therapeutic approaches and, aims to bridge the existing knowledge gap that will assist in overcoming challenges in HGG.

## 1. Introduction

Cancer is categorized by the World Health Organization (WHO) as the second deadliest disease, with an estimated death of 9.6 million globally in 2018 [1]. According to Siegel et al., in the United States alone, the number of newly diagnosed cancer patients shows an increment of around 8.94%, with an increase of 2.90% in mortality rate in the last five years [2,3]. According to the WHO classification, glioblastoma (GBM) is a grade IV glioma and the most aggressive form of diffuse glioma belonging to the astrocytic lineage. Out of all gliomas and primary brain tumors, GBM makes up the majority of it. This makes it the most common primary brain tumor [4].

Gliomas are neuroepithelial CNS tumors that can be classified into low-grade gliomas (LGG) and high-grade gliomas (HGG) [5]. Gliomas are characterized by the grade of malignancy, morphological characteristics, and molecular markers alteration based on the 2016 WHO classification [6]. Grade II–IV glioma includes astrocytoma, oligodendrogliomas, and glioblastoma (GBM) [6,7,8]. Although the prevalence of HGG (4.55%) is low compared to other cancers, it remains a fatal and aggressive type of primary brain tumor based on the CBTRUS statistical report from 2009–2013 in the US population [2,3,9]. LGG patients often respond better to treatment and have a better prognosis though patients may experience relapse with more aggressive glioma features [10,11]. GBM is the most aggressive adult form of HGG, accounting for 60–80% of all incidence among elder individuals (median age of diagnosis of 62 years old) with a median survival of 15 months [4,12,13]. However, many HGG patients are not well suited for oncological treatment and are referred for palliative care instead [4,13,14,15].

The recommended standard of care for newly diagnosed HGG includes surgical resection, radiotherapy, and chemotherapy. Despite the optimal primary treatment, the patients’ prognoses remain abysmal. According to the Central Brain Tumor Registry of the United States, the median overall survival is between 15–23 months and a low five-year survival rate (between 2007–2011) [4,13,16]. This can be due to surgical resection’s inefficacy to fully resect the tumor and lack of effective therapeutic approaches to exclusively target HGG tumors that often have high tumor heterogeneity with complex tumor microenvironment [17]. Currently, there are no curative treatment options available for HGG, especially in GBM, and the current therapeutic leads to adverse side effects. Recent clinical trials utilize targeted treatment like immunotherapy and gene therapy as an adjuvant to counter the impact of immune dysregulation by stimulating the patient’s immune system [18]. Recent breakthroughs in unraveling the molecular pathogenesis in HGG would improve the classification of gliomas, determine a patient’s prognosis, and develop a therapeutic regimen based on each patient’s requirement.

Additionally, research has looked into the potentiality of natural products as nutraceutical-based adjuvants [19,20,21]. Hence, this present review aims to discuss the current views of drug development and therapy in HGG. Additionally, this review discusses the therapeutic potential and the challenges associated with each of the different treatment modalities. The highlights and discussion in this review aim to improve the existing knowledge and bridge the gap in HGG research and advancement, particularly in the last decade. We hope this will provide a more comprehensive understanding of the development of more precise, effective, and personalized therapy in HGG patients.

## 2. Overview of Standard Therapy in HGG

### 2.1. Surgical Resection

Surgical resection is regarded as the benchmark to alleviate symptoms due to tumor mass. It decompresses the bulk of the tumor, reduces the elevated intracranial pressure, and provides a sufficient histological analysis of the tissue sample [22]. GBM’s residual presence is often seen in tumor recurrence cases due to their highly infiltrative and proliferative nature. Therefore, maximizing the tumor removal, which includes excising the margins with minimal impacts on the healthy surrounding tissue, is crucial to improve the life expectancy of GBM patients [23]. The average survival for patients who have undergone surgical resection only instead of biopsy is significantly higher (7 months vs. 3.5 months), according to Lara-Velazquez et al. [24]. Thus, the degree of tumor resection influences GBM patients’ prognoses. Although radical extirpation is usually the aim, this is not attainable due to the infiltrative nature of GBM cells [8,24,25]. Hence, every neurosurgeon’s realistic aim is to resect up to a 90% threshold without causing surgery-related neurological deficits. The innovations in the field of neurosurgical oncology which can aid in ensuring maximum cytoreduction are summarized in Table 1.

### 2.2. Chemotherapy

**Figure 1:**
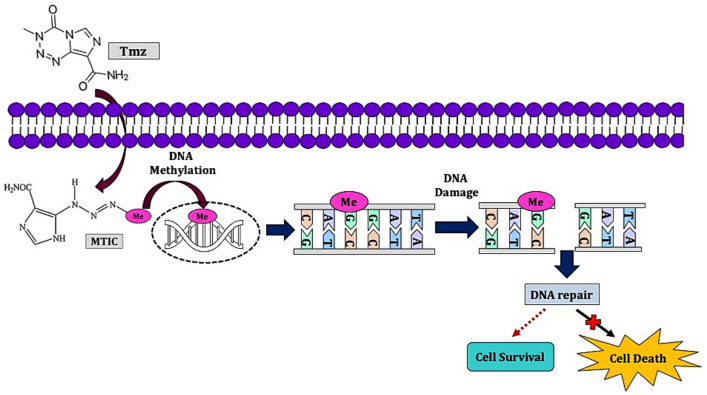
Schematic depiction of Tmz mode of action. Tmz undergoes spontaneous hydrolysis intracellularly to form monomethyl triazene 5-(3-methyltriazen-1-yl)-imidazole-4-carboxamide (MTIC). MTIC then hydrolyzed to form 5-aminoimidazole- 4-carboxamide, which later converts into methylhydrazine [52]. Methyldiazonium, an active cation, then methylates the nucleobases, preferentially N^7^ position of guanine (N^7^-MeG; 70%), guanine rich site and to a certain extend at N^3^ adenine (N^3^-MeA; 9%) and O^6^ guanine residues (O^6^-MeG; 6%) [59,60]. This results in the formation of nicks in the DNA resulting in apoptosis and cell cycle arrest at the G_2_/M phase [60,61].

The common alkylating agents used in HGG are temozolomide (Tmz, 8-Carbamoyl-3-(2-chloroethyl)imidazo (5, 1-d)-l,2,3,5-tetrazin-4(3 H)-one) (Figure 1) and lomustine (chloroethyl-cyclohexyl-nitrosourea, CCNU) [40,41,42]. Before Tmz, CCNU was the first-line of treatment in GBM patients (110 mg/m^2^ orally every six weeks) [43]. Currently, CCNU is administered in recurrent GBM patients [41,42,44]. CCNU is highly lipophilic, enabling BBB penetration, making it an ideal candidate in GBM and treating other HGGs [40,44]. CCNU induces alkylation of DNA and RNA strands resulting in the formation of O6-chloroethylguanine lesions [44]. CCNU inhibits the enzymatic function of key enzymes involved in the carbamoylation process of amino acids, interfering with transcription and translation processes [45,46,47]. CCNU efficacy in GBM relies on *MGMT* and mismatch repair status, which repair the interstrand links form via CCNU toxicity [44,48]. Although GBM patients with methylated *MGMT* and deficient mismatch repair often have a better prognosis with CCNU, the six-months progression-free survival (19%) and median overall survival (7.1 months) remains low, particularly in recurrent GBM patients as demonstrated in phase III clinical trial [49].

Tmz had become the major game-changer in HGG, replacing nitrosourea-based chemotherapy following a phase II randomized trial for recurrent GBM [50]. Tmz is hydrophilic and small in size (194 Da) with BBB’s efficient penetration. Oral administration of Tmz is accompanied by 100% bioavailability in the blood flow [51,52,53]. However, in brain tumor tissue, the concentration of Tmz is around 20% of the plasma level [54,55]. The cerebrospinal fluid concentrations are similar, but the levels can rise to 35% of plasma levels [54,56]. Tmz is stable in acidic conditions and labile in an alkaline state with a plasma half-life of 1.8 h at pH 7.4 [57]. Moreover, brain tumors have a higher alkaline pH compared to the surrounding healthy tissue, a condition that favors Tmz prodrug activation [57]. Moreover, Tmz demonstrates an acceptable safety profile with mild or moderate adverse effects making it a standard treatment in recurrent HGG while CCNU being the second-line therapy [50]. However, Tmz is associated with side effects such as nausea, fatigue, significant myelosuppression, thrombocytopenia, severe infections, and myelodysplastic syndrome [54,58].

The standard dose (75 mg/m^2^/day) and concurrent administration of Tmz in recurrent anaplastic astrocytoma patients demonstrated 6-month progression-free survival and overall survival of around 46% and 13.6 months, respectively [62,63,64]. The continuous dose-dense Tmz for recurrent anaplastic astrocytoma can help overcome the drug resistance by decreasing *MGMT* activity with anti-angiogenic properties [62,65,66]. In contrast, anaplastic oligodendroglioma patients are responsive to various chemotherapy such as PCV (procarbazine, vincristine, lomustine) and Tmz [62,67]. Although PCV administered prior or after radiotherapy did not improve the overall survival among newly diagnosed low-grade anaplastic oligodendroglioma patients, significant improvement was observed in the progression-free survival when PCV administered following radiotherapy (24.3 months vs. 13.2 months) [67,68,69]. This improvement, however, demonstrated significant toxicity and patients’ low quality of life. Interestingly, high-grade anaplastic oligodendroglioma patients with 1p/19q co-deletion treated with radiotherapy only or a combination of radiotherapy and PCV exhibited improvement in the overall survival [70,71,72,73]. Tmz is more tolerable than PCV, and recent clinical trials supported its use for anaplastic oligodendroglioma patients with intact 1p/19q and wild-type *IDH1* [68,71]. Tmz demonstrated a positive response in anaplastic oligodendroglioma patients and is used as the first line treatment in progressive or recurrent anaplastic oligodendroglioma who are CT-naïve [74,75]. Previously, in a prospective GICNO study, it was reported that co-deletion of 1p/19q is associated with Tmz responses, and *MGMT* methylation is correlated with co-deletion of chromosome 1p/19q in anaplastic oligodendroglioma [76]. Hence, *MGMT* methylation and 1p/19q co-deletion could confer a favorable prognosis in patients with HGG. Thus, a complex model integrating 1p/19q co-deletion, *MGMT* methylation, *IDH1* mutations while taking into consideration the patient’s age and histopathological diagnosis should be integrated to validate this [77]. The univariate analysis of the NRG Oncology/RTOG 0424 trial also validated *MGMT* promoter methylation as an independent prognostic biomarker, particularly in LGG patients receiving a combination of Tmz and radiotherapy [78]. This analysis demonstrated a significantly reduced OS association with unmethylated *MGMT* promoter status. Additionally, this study highlighted *MGMT* promoter methylation as a potential prognostic tool besides *IDH1/2* mutation for LGG [78]. Anaplastic oligodendroglioma patients treated with radiotherapy and PCV obtained an approximately objective response rate of 44% towards Tmz with the median overall survival of 10 months [74,75,79,80].

Typically, the standard treatment for newly diagnosed GBM involves a four-pronged approach. Following surgery and histopathological and molecular diagnosis, patients are subjected to radiotherapy with concurrent administration of Tmz [81]. Stupp and coworkers [82] showed that patients who received radiotherapy with concomitant daily Tmz followed by six cycles of adjuvant Tmz recorded an improved median survival (14.6 months) as compared to control groups (12.1 months). Additionally, the patients showed a 26.5% improvement in the two-year survival rate compared to the traditional approach (10.4%). For patients over 70, where surgery is not an ideal option, less aggressive radiation or Tmz treatments are prescribed [83]. Nevertheless, due to tumor resistance over time, extreme neurological deterioration, and the high risk of relapse, these therapies are frequently proven ineffective [60,84].

### 2.3. Radiotherapy

Although radiotherapy following surgical resection does not offer complete curative effects in most HGG cases, it offers progression-free survival benefits compared with chemotherapeutic agents [16,69]. In a prospective study (NOA-04 study), initial chemotherapy (Tmz or Vincristine) combined with deferred radiotherapy was equivalent to using radiotherapy alone [85]. Additionally, the study showed no significant difference in progression-free survival between patients who received chemotherapy versus initial radiotherapy. This study also indicated that *IDH1* mutation has a favorable prognosis than the methylation of *MGMT* promoter or 1p/19q co-deletion. Hence, anaplastic astrocytoma with *IDH1* wild-type and *MGMT* methylation patients may be more suitable treated with chemotherapy and if the *MGMT* is unmethylated, they are better treated by radiotherapy only. This is because *MGMT* encodes for a DNA repair enzyme that interferes with DNA alkylation by Tmz [86]. Additionally, when the CpG islands located in the promoter regions of *MGMT* are methylated, it suppresses *MGMT* transcription. Hence, individuals with methylated *MGMT* HGG exhibit a favorable response when given Tmz [86]. Re-irradiation is also useful in providing palliative benefit and is considered safe in recurring anaplastic astrocytoma patients [62,87,88].

In three clinical trials, a combination of radiotherapy with PCV in anaplastic oligodendroglioma patients (EORTC 26955 and RTOG 9402) and LGG (RTOG 9802) demonstrated an improvement in overall survival [62]. In another study, PCV addition to radiotherapy in anaplastic oligodendroglioma patients is not restricted to tumors with 1p/19q co-deletion but also to *ATRX* and *IDH* mutations [89]. Anaplastic astrocytoma patients may share similar molecular traits with anaplastic oligodendroglioma patients having 1p/19q co-deletion and low-grade astrocytoma with *IDH* mutations. The results from these clinical studies can be extrapolated for all diffuse gliomas, including anaplastic astrocytoma. The efficacy of Tmz in combination with radiotherapy in treating anaplastic astrocytoma with 1p/19q co-deletion yielded superior results as opposed to radiotherapy only [63,90]. In 2017, the European Union of Neuro-Oncology suggested maximal safe resection followed by radiotherapy only or chemotherapy only (Tmz or PCV) for individuals with newly diagnosed anaplastic astrocytoma lacking 1p/19q co-deletion [40]. Postoperative radiotherapy (total dose of 60 Gy across 30 fractions) is commonly given in anaplastic oligodendroglioma patients [91,92,93]. However, there are different views that radiotherapy is unnecessary for anaplastic oligodendroglioma patients with 1p/19q co-deletion due to neurocognitive impairment. Nevertheless, there is no substantial scientific evidence of this opinion, and therefore, radiotherapy is still considered the standard therapy for all malignant gliomas until further evidence is made available.

## 3. Challenges in HGG Standard Therapy

Though HGG therapy gives the patients an extended overall survival, it comes with an actual impedance [94]. For example, in oligodendroglioma, ~4% of the cancer stem cells (CSC) are cycling stem cells that promote tumor growth and recurrence. In comparison, the remaining 96% are non-cycling cancer cells that are resistant to chemotherapy and radiotherapy [94,95,96]. Mutation of *IDH* renders cells incapable of fully utilizing the citric acid cycle, which creates ATP deprivation, leading to a low cell cycle performance [94]. Although chemo-radiotherapy has substantial benefits in prolonging the median overall survival (>14 years), even after the prescription has been repealed by six rounds and the dose has been lowered, the mortality rate remains high. In RTOG 9402 and EORTC 26951 clinical trials, patients prescribed with lesser cycle therapy and lower dose exhibited significant hematological toxicities (56% and 46%, respectively) [97]. These toxicities further necessitate the development of more effective therapy that selectively targets tumor cells while maintaining patient quality of life.

In anaplastic oligodendroglioma patients, radiation is included as post-surgery initial treatment. However, the PCV regime has been added as part of disease management (based on EORTC 26951 and the RTOG 9402 trials), which demonstrated prolonged survival and better radiographic response rate (93–100%) in 1p/19q co-deletion gliomas than Tmz (35–82%) [98]. However, the combination of PCV with radiotherapy has been associated with cognitive deterioration and brain damage due to prolonged irradiation. In the hope of sparing and delaying such damage, the possibility of including only PCV chemotherapy has been suggested as an alternative option [98]. Although PCV was suggested as the potential standard care chemotherapy based on the EORTC/RTOG (phase III) trial (PCV + radiotherapy) in patients with 1p/19q codeletion, there is still an ongoing debate on Tmz use as a replacement for PCV due to its lower toxicity and easy administration mode [98,99,100]. Although PCV demonstrated better effects than Tmz, the NOA-04 trial demonstrated no difference between PCV and Tmz in combination with radiotherapy. Suggesting neither regimen is superior to the other [101]. An ongoing two-arm phase III clinical trial (NCT00887146) is looking into the direct comparison between PCV- radiotherapy combination against concomitant and adjuvant Tmz with radiotherapy anaplastic oligodendroglioma patients with 1p/19q co-deletion [102].

Anaplastic astrocytoma patients with 1p/19q co-deletion and *IDH* mutation often have a better prognosis. In contrast, patients with only *IDH* mutation and intact 1p/19q have moderate prognoses [62]. Although wild-type *IDH* anaplastic astrocytoma patients tend to have poorer prognoses, they share similar molecular alterations with GBM patients, including *EGFR* amplification, gain in chromosome 7, and loss in chromosome 10 [103]. In *IDH*-wild type astrocytoma, the high tumor heterogeneity further under defined treatment strategy [104]. Hence, patients are diagnosed and treated on a case-to-case basis based on age, Karnofsky Performance Status (KPS), loss in chromosome 10, and gain in chromosome 7 along with the clinical and radiological course, and *MGMT* methylation status [104]. Although *IDH*-mutated diffuse glioma patients have better prognoses and higher sensitivity to chemotherapy, the IDH protein may represent a druggable antigen [105]. IDH catalyzes the conversion of α-ketoglutarate into 2-HG, causing D-2-HG accumulation, which can inhibit numerous histone demethylases. The D-2-HG acts as a competitive inhibitor towards α-KG-dependent histone demethylases [106]. Additionally, D-2-HG also competitively inhibits the function of ten-eleven translocation methylcytosine dioxygenase 1 and 2 (TET 1 and TET2). TET functions as a catalyst for 5-methylcytosine (5-mC) demethylation process through a series of conversions. However, when D-2-HG is present, it limits the ability of cytosine to demethylate. Hence, this causes 5-mC to accumulate in the genome, which induces cytosine demethylation [107]. Increased histone methylation associated with D-2-HG can restrict cell differentiation which is vital in gliomagenesis and cell maintenance [108]. Furthermore, D-2-HG may affect numerous pathways involved in DNA repair. It inhibits the α-KG-dependent alkB homolog (ALKBH) enzyme, which sensitizes cancers with *IDH* mutations to DNA alkylating agents [109]. Moreover, mutation to *IDH1* downregulates the ataxia-telangiectasia-mutated (ATM) signaling pathway via an alteration to histone proteins’ methylation [110], resulting in enhanced sensitivity towards agents that damage the DNA. Moreover, *IDH* mutation causes a reduction in NAD+, affecting the poly (ADP-ribose) polymerase-1 (PARP1)-associated DNA repair pathways [107].

The current standard protocol of treating HGG can be improved using immunotherapy or gene therapy to target the DNA repair pathways. Furthermore, decreased glutamate and enhanced glutaminolysis are commonly seen in cancers with *IDH* mutation. Hence, inhibiting glutaminases, would suppress *IDH* mutant cancers from growing as decreased glutamate and dependence on glutaminolysis are important characteristics of *IDH* mutant cancers [107].

Up-to-date chemotherapy of either PCV or Tmz, depending on physicians/patients’ preference for residual tumor patients after initial surgery, is recommended either with radiotherapy for diffuse astrocytomas (*IDH* mutated or wild type) or alone for oligodendrogliomas [111]. Although prolonged survival of oligodendroglial patients over anaplastic astrocytomas was reported, the differences were not statistically significant [112]. The lack of details on possible allelic losses on chromosomes 1p/19q and *IDH* mutation status in the patient population prevents a full assessment of observing survival disparity after radiotherapy [112].

**Figure 2:**
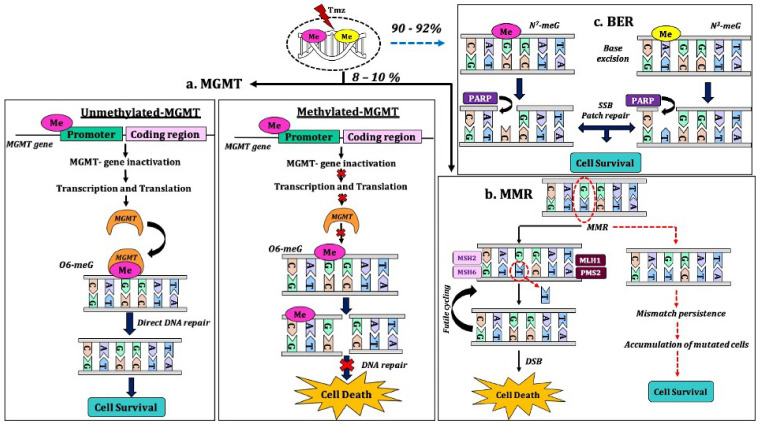
Mechanisms of Tmz resistance. (**a**) The expression of *MGMT* along with successful DNA repair mechanisms: (**b**) mismatch repair; (**c**) Base excision repair resulting in survival as GBM tumors leading to chemoresistance.

Tmz efficacy within a tumor can be affected by DNA repair systems (Figure 2) such as base excision repair, mismatch repair, and notably, the methylation status of *MGMT* [54]. *MGMT* encodes O6-alkylguanine-DNA alkyltransferase (AGT) protein that removes the alkyl genotoxic O6-meG adducts leading to chemoresistance [113]. In GBM, the therapeutic advantage is most effective in 50% of patients whose tumors exhibit *MGMT* promoter methylation [54,113]. GBM patients who initially respond to Tmz eventually experience a relapse before or after treatment termination [114]. Cysteine-phosphate-guanine (CpG) is the DNA methylation site of the *MGMT* gene that renders its inactivation leading to reduced gene expression. Within this promoter region, 97 CpG loci are present with two different methylation domains. However, not all the methylation site of CpG loci regulates *MGMT* expression [115,116,117]. An unmethylated promoter region corresponds to an active *MGMT* gene leading to an increased expression commonly associated with Tmz resistance [117,118,119,120,121]. However, *MGMT* accounts for only 8–10% of Tmz resistance in GBM [119,122]. Although *MGMT* promotes Tmz resistance, additional factors such as post-translational modifications on histones proteins [123] and miRNAs deregulation [124] are also involved.

The mispairing of O^6^-methylguanine (O^6^-MeG) with thymine induced by Tmz is seen during the replication of DNA in unmethylated *MGMT* cells (Figure 2) [125,126]. This mispairing results in the mismatch repair system’s activation to excise thymine from the newly synthesized daughter strand, leaving O6-MeG the parental strand intact. This restorative process undergoes repetitive cycles by reinsertion and removal of thymines leading to cell cycle arrest and apoptosis [125,126]. Impairment in mismatch repair system contributed by gene mutations such as *melanocyte-stimulating hormone 2* (*MSH2*), *MSH6*, *mutL homolog 1* (*MLH1*), and *post-meiotic segregation-increased Saccharomyces cerevisiae 2* (*PMS2*) [57,59,122]. In a study by McFaline-Figueroa and colleagues [127], Tmz showed modest deregulation in the expression of MutS ∝ MMR recognition complex components with MSH6 (50%) and MSH2 (70%) proteins. The observation is correlated with the diminished mismatch repair activity and accounted for Tmz resistance. However, these mutations are predominant among recurrent patients with methylated *MGMT* GBM than primary GBM, suggesting that initial Tmz sensitivity may exert selective pressure to alter mismatch repair protein expression [57,59,122].

Base excision repair system is involved in repairing DNA damage caused by oxidizing, ionizing radiation, or alkylating agents [119]. The methylation of N7-guanine (60–80%) and N3-adenine (10–20%) represents more than 90% of the methylation by Tmz and is rapidly repaired by base excision repair [119,122]. When one or more base excision repair components are mutated, its ability is deficient and contributes to Tmz cytotoxicity [128,129]. Notably, N3 lesions are lethal if not repaired, as opposed to N7 lesions, which leads to inhibition of PARP-1. Such inhibition results in the accumulation of DNA nicks, which is removed via the cell death mechanism. DNA damage causes hyper-activation of PARP-1, resulting in NAD+ and ATP depletion, leading to cell death [59,122]. Although N7-guanine and N3-adenine methylation are higher than that of O6-guanine, base excision repair role in Tmz resistance is reportedly less critical than that of *MGMT* and MMR mutation [59,122]. Current studies have found that ferroptosis, a novel cell death mechanism, has been linked to cancer progression and drug resistance in GBM [130,131]. Although ferroptosis’s role in Tmz resistance may serve as a potential therapeutic avenue in sensitizing GBM cells to Tmz, further studies are needed to fully understand its mechanism.

Generally, Tmz can induce cell cycle arrest and apoptosis via DNA damage in tumors that lack *MGMT*. However, in glioma, such as U87 cell line, they can develop a response against Tmz-induced apoptosis and arrestment of the cell cycle at the G_2_/M phase [119,132]. Such finding suggests the possible involvement of other cell death mechanisms. Recent studies have shown Tmz treatment induces autophagy in GBM cells. Chemotherapeutic agents and radiations are known to activate autophagic pathways in cancer cells [119,133]. However, autophagic cell death is controversial as its dual effect includes pro-survival or pro-death response [134,135]. Autophagy is a cytoprotective mechanism that can provide cells with energy, prolonging their survival and evading apoptosis [134]. In cancer cells, such a process can be detrimental as it favors the survival of cancer cells contributing to chemoresistance. For instance, oxidative stress induced by chemotherapeutic drugs enables cancer cells to survive, even in hypoxic and nutrient-deficient environments [134]. Autophagy through ATM/AMPK pathway can result in the formation of acidic vesicular organelles and aggregation of LC3, which are vital for cytoprotective and cell survival [136,137]. Additionally, the hypoxic microenvironment in HGG tumors is a vast challenge in radiotherapy as it induces radioresistance [138,139]. In GBM, the hypoxic conditions can induce stemness and upregulate *MGMT* expression [59,140,141,142]. This hypoxic condition elevates HIF-1, which increases glycolysis, pentose phosphate pathways, and serine production pathways, heightening antioxidant production, thereby buffering ROS’s actions induced by radiation. Furthermore, the hypoxic state increases ROS production, stimulating an antioxidant generation loop [143,144,145]. Hyperbaric oxygen is used to counteract the tumors’ hypoxia [146,147]. GBM tumors subjected to hyperbaric oxygen displayed reversal/reduced radioresistance, chemoresistance, and radiation-enhanced tumor motility [147,148,149,150,151]. Radiation from radiotherapy can adversely affect the patients’ neurocognitive ability [152,153,154]. Hence, the application of fractionated radiotherapy or interstitial brachytherapy is thought to be safer and well-tolerated among HGG patients [155,156,157,158,159]. In a retrospective study, 59 recurrent GBM patients demonstrated prolonged median survival by eight months when given a median dose of 36 Gy radiotherapy with 2 Gy given each day [160]. However, there are insufficient data for fractionated radiotherapy to be used routinely in the setting of recurrent GBM. Like fractionated radiotherapy, brachytherapy also enables a sharp dose gradient by placing a radiation source within the tumor volume to be treated [161]. This is usually carried out postoperatively, and it is offered to patients with a good performance status and more resectable small tumor in volume. One of the approaches in which brachytherapy can be utilized is by placing permanent iodine 125 (I-125) seeds in the resection cavity [161]. In a retrospective study by Darakchiev et al. in 2008, utilizing brachytherapy in patients with GBM reported a favorable result with the median survival of 15.9 months [162]. However, the disadvantage of brachytherapy is the high incidence of radionecrosis. Hence, brachytherapy has to be used with caution [161].

## 4. Drug Development for HGG: Advancements and Challenges

### 4.1. Gene Therapy

Alterations in various genes largely drive tumorigenesis. Thus, gene therapy can be utilized to inhibit the oncogenic properties of tumor cells [163,164]. Gene therapy in cancer involves introducing a tumor-suppressing or growth-regulating gene into the tumor [163]. Since conventional treatment modalities are incapable of overcoming resistance, the genetic component of tumor cells may be manipulated by utilizing gene therapy to acquire a therapeutic benefit. To improve the delivery of these therapies, delivery vectors such as viral vectors, polymeric nanoparticles, and non-polymeric nanoparticles have been studied [163,165,166,167]. Although the use of these viral and non-viral vectors offers therapeutic advantages, their utilization in HGG possesses some challenges (Table 2 and Table 3).

Viruses target specific cells and hijack the cell’s replicative properties. In doing so, this leads to the release of numerous copies of the virus and the host cell’s death [168]. This specific capacity of viruses allows them to selectively attack and overwhelm a particular tumor cell population while sparing the other surrounding cells in its vicinity, making viral therapy a potential candidate for the treatment of HGG [168]. Various trials have been conducted to assess their efficacy, summarized in Table 4 [168]. According to a meta-analysis carried out by Artene et al. in 2018 (Table 4), viral therapy improved the overall-survival among newly diagnosed HGG patients (HR = 0.72, 95% CI: 0.54–0.97) [168]. However, the meta-analysis findings stated these studies were not statistically significant (*p* = 0.13). Additionally, viral therapy did not statistically improve the progression-free survival [168]. Hence, gene therapy using viral agents alone may not be a feasible treatment modality in HGG.

Glioma cells secrete immunosuppressive factors that prevent them from being detected and eliminated by the immune system. Additionally, glioma cells can express CD95 ligand on their surface, which allows them to trigger apoptosis and subsequently reduce T-cells’ infiltration in the tumor microenvironment [169,170]. Therefore, researchers focus on developing multitarget therapies that enhance tumor detection and clearance, promoting cell death such as apoptosis, while reducing processes such as angiogenesis and chemoresistance. Such therapies include the use of immune therapy, electric field therapy, nanoparticles and phytochemicals that can further enhance Tmz and radiotherapy efficacy.

**Table 2:** Example of viral vectors in HGG studies.

Vector	Findings
Herpesvirus and Retrovirus	The use of herpes simplex virus as suicide gene therapy by converting antiviral drugs which prolonged prodrug treatment, improved survival and inhibited proliferation as well as tumor growth [171,172,173].
	TOCA 511 resulted in the promotion of T cell expansion (Th1, Th2 in CD4^+,^ CD8^+^), mediated antitumor immune response, and concentrated the effect of drugs at the tumor site which increased direct tumor cell death, alterations in immune cell infiltration, and improved survival [174,175,176,177,178].
	Retroviral replicating vectors (RRV) based on gibbon ape leukemia virus enabled high-efficiency gene transfer and persistent expression of *E. coli* nitroreductase prodrug activator genes, resulting in efficient cell killing, suppression of tumor growth, and prolonged survival upon CB1954 administration [166].
	Semi- and pseudotyped-RRV system harboring two suicide genes—HSV1 thymidine kinase and yeast cytosine deaminase and prodrug demonstrated high oncolytic capability against extremely heterogeneous and treatment-refractory GBM which promoted the inhibition of cell proliferation, angiogenesis, increased apoptosis, and the depletion of tumor-associated macrophages in orthotopic GBM [179].
Adenovirus	The replication-deficient adenovirus mutant thymidine kinase (ADV-TK) in combination with ganciclovir improved recurrent patients’ survival, integrin antagonist cRGD (EMD121974) promoted adenovirus-mediated REIC/Dkk-3 reduction of cell proliferation and mice survival. Adenovirus is also used to transfect *p53* gene, mediated cytotoxic immune therapy of prodrug and PTEN, PI3K inhibitors [180,181,182,183].

**Table 3:** Benefits and challenges of viral and non-viral vectors in HGG [163].

Vector	Benefits	Challenges
Adenovirus	Deliver large amounts of DNA	The gene expression is transient Elicits an immune response against the tumor cells
Adeno-associated virus	Can transfer genetic material to non-dividing and dividing cells	Producing vectors is difficult The transgene capacity is limited Elicits an immune response
Retrovirus	Can transfer genetic material to cells that are dividing The expression of the vector is sustained	Elicits an immune response Unable to transfect non-dividing cells Low transfection efficiency in vivo Risk of insertion at the wrong location
Gold nanoparticles	Can be used to treat and image the tumor Can be functionalized for targeting	Non-biodegradable
Polymeric micelles	Can be functionalized for targeting It is self-assembled with nucleic acids	Increased the cytotoxic effects for poly(ethylenimine) as well as other cationic polymers. Low loading ability
Dendrimer and Dendrigraft	It is self-assembled with nucleic acids Can be functionalized for targeting Non-immunogenic	Increased cytotoxicity for cationic dendrimers Limited release of therapeutics
Poly(β-amino ester)	Biodegradable Compared to other cationic polymers, it has a lower cytotoxic level Its efficiency to transfect is high	It has limited control when releasing the therapeutic agent.

**Table 4:** Studies that utilized viral therapy in the treatment of HGGs. (AA—anaplastic astrocytoma, AO—anaplastic oligodendroglioma, GBM—glioblastoma, OS—overall survival, PFS—progression-free survival) [168].

Study Reference	WHO Classification of Tumor	Phase of the Clinical Trial	Total Patients		Outcome
			Experimental Group	Placebo Group	
Rainov et al. [184]	IV (GBM)	III	111	103	OS, PFS
Stragliatto et al. [185]	IV (GBM)	I/II	22	20	OS, PFS
Westphal et al. [186]	IV (GBM)	III	119	117	OS
Wheeler et al. [187]	III (AA,AO), IV (GBM)	Ib/IIb	48	134	OS, PFS

### 4.2. Immunotherapy

Immunotherapy is used to treat many cancers, such as melanoma, renal cell carcinoma, lymphoma, and non-small lung cancer [188]. Immunotherapy research is still ongoing to explore potential newer target sites in HGG (Table 5) [18]. The treatment modalities that can render tumors more vulnerable to one’s immune system are considered strong candidates. Dendritic cell (DC) vaccine can serve as a mediator between the innate and adaptive immune systems by processing and presenting the antigens to either B or T-cells. This will then trigger an immune response via T or B-cells [168]. Thus, this makes them an appealing vaccine candidate that can induce an immune response against tumors [168]. Various trials have been conducted to assess their efficacy, summarized in Table 6 [168].

According to a meta-analysis by Artene et al. (Table 6), DC therapy prolonged the overall survival of newly diagnosed (HR = 0.65, 95% CI: 0.45–0.93, *p* = 0.02) and recurrent HGG patients (HR = 0.63, 95% CI: 0.46–0.88, *p* = 0.006) [168], Also, the newly diagnosed HGG patients had a 51% chance of having a longer progression-free survival period between treatment initiation and the confirmation of tumor recurrences via MRI. Despite this, the results were insignificant (*p* = 0.10) [168]. In conclusion, the meta-analysis by Artene et al., exhibited that DC therapy provided significant improvement in the overall survival among both groups of patients (newly diagnosed and recurrent HGG) [168]. However, all the studies included are in Phase I or II, which have limited value statistically. Hence, larger phase III trials are required to justify this treatment modality further. In a randomized phase III clinical trial (NCT00045968), the addition of DCVax to regular therapy (Tmz) in newly diagnosed GBM patients prolonged the two and three-year survival rate by 66.7% and 46.4% respectively in patients with methylated *MGMT*, whereas in patients with unmethylated *MGMT,* the two and three-year survival rate is 32.1% and 11% respectively [189]. The authors concluded that the addition of DCVax-L to standard therapy is safe and feasible for patients with GBM and may prolong their survival.

Monoclonal antibodies (Mabs) have high affinity and specificity in targeting growth factor receptors such as PDGFR, VEGFR, and EGFR. One challenge of utilizing Mabs is that they may not easily cross the BBB due to their large molecular size. Hence, to overcome this, Mabs can be attached to a nanocarrier surface via a pre-adsorption process and prevent biomolecular corona formation [190,191]. The nature of HGG, mainly GBM cells, which are incredibly heterogenic, makes the usage of monovalent vaccines inadequate to control tumor progression [192,193,194]. For example, some peptide vaccines are vastly restricted towards the *EGFR_V_III* variant, which is only present in 23–33% of GBM patients [195,196]. Thus, in GBM patients without this variant, the peptide vaccines may be futile. Moreover, even if some patients have the *EGFR_V_III* variant, the natural evolution of the GBM tumor could result in a loss of this variant subtype, thus, causing peptide vaccines to be ineffective, as seen in phase III of the ACT IV trial [197]. One strategy is to use a polyvalent vaccine so that a larger population of tumor cells can be targeted.

CAR T cell therapy, which utilizes engineered T cells to kill tumors by targeting cell surface-specific antigens, has gained emerging interest in preclinical and clinical GBM studies [198,199,200,201,202]. Moreover, single use of CAR T cell therapy has demonstrated tolerable safety profiling and feasibility in glioma. For instance, the use of CD70-specific CAR T cells, which recognize CD70 positive GBM in vitro, promotes tumor regression in the xenograft and syngeneic GBM models [203]. CD70 expression is generally associated with poor survival among *IDH* wild-type primary LGGs, the mesenchymal GBM subtypes, and the recurrent GBM patients. In a study by Tang and coworkers, CAR T cells’ construction, which targets B7-H3 was delivered using lentivirus in preclinical primary and GBM cell lines [204]. B7-H3 is highly expressed in glioma patients as it is linked to tumor malignancy and poorer survival. Using the constructed CAR-T-cell-B7-H3 targeting, they demonstrated antitumor and cytotoxic activities, which promoted longer median survival in the orthotropic GBM models.

A number of phases I and II clinical trials have shown the efficacy of CAR T cell therapy in GBM patients (targeting IL-13Rα2, *EGFRvIII*, EphA2, and HER2) [199]. However, these molecular targets are more prone to antigen escape since they are not homogenously expressed in GBM tumors. Additionally, the high tumor heterogeneity and complex GBM tumor microenvironment serve as limitations. These situations may impede the CAR T cell migration towards the GBM tumor site and affect its persistence. Thus, a one-fit-target approach may not be suitable. Additionally, combining immunotherapy such as CAR T cell therapy with other therapeutic approaches could confer greater efficacy. In a recent study, the addition of TGFβ-trap into *EGFRvIII*-specific CAR T cell further prolonged the survival of mice [205]. The authors also observed the elevated expression of M1 polarization markers of GBM-infiltrated microglia, which may be responsible for disrupting the immunosuppressive tumor microenvironment. In a study by Bielamowicz and colleagues [206], the use of trivalent CAR T cells (UCAR T cells) could be beneficial in overcoming antigenic heterogeneity in GBM. In this cohort study, co-targeting HER2, IL13Rα2, and EphA2 overcomes the interpatient variability and activates the immune synapses to improve cytotoxicity and release of cytokines when compared to monospecific and bispecific CAR T cells. Additionally, the low concentration of the UCAR T cells enhances the control of established autologous GBM patient derived xenografts and promotes animal survival. In a different study, the local GBM tumor irradiation resulted in a synergistic antitumor of natural killer group 2-member D (NKG2D) CAR T cell therapy in immunocompetent GBM mice [207]. The tumor irradiation enhances the NKG2D CAR T-cell activity, tumor recognition, and better trafficking of the intravenous injected NKG2D CAR T cells.

The therapeutic efficacy of immunotherapy such as CAR T cell, peptide vaccine, or monoclonal antibodies can be improved by (i) combining them with the existing conventional therapy, (ii) the use of multitarget agent such as natural products, and (iii) the construction of multi-target CAR T cells. In a study by Suryadevara and coworkers [208], preexposure of GBM tumors to Tmz promotes *EGFRvIII* CAR T cells’ efficacy. The authors demonstrated that the *EGFRvIII* CAR T cell’s engraftment would benefit from Tmz-induced lymphopenia, which extended the survival of the animal models. Their study suggested using standard therapy such as TMZ as a first-line approach or preconditioning before the systemic infusion of *EGFRvIII* CAR T cell. Following these observations, the authors conducted a phase I trial on 12 newly diagnosed GBM patients subjected to Stupp regimen and three cycles of dose-intensified Tmz before administering *EGFRvIII* CAR T cell (NCT02664363).

However, the combination of immunotherapy with other therapeutic approaches may also heighten the toxicity and adverse effects. Therefore, such an approach of combining CAR T cells, immune checkpoint blockades, monoclonal antibodies, conventional therapy, and natural products still requires phase I and II clinical trials (which some are undergoing) to provide important safety information. Additionally, these clinical trial data are essential in limiting or superimposing the toxicities while justifying the efficacy and potential pitfalls. To date, most of the studies of CAR T cell and its combination with other therapies are mostly focusing on preclinical and xenograft of immunocompromised GBM models. This does not represent the complex tumor microenvironment of GBM. Thus, it will be important to evaluate CAR T cells and other therapy combinations in immune-competent GBM models. Additionally, the use of 3D culture and patient-derived xenografts would be beneficial as they closely mimic the tumor microenvironment and phenotypic of GBM.

**Table 5:** Immunotherapy in HGG.

Immunotherapy	Description
Bevacizumab	Promotes survival, enhances standard therapy, and inhibits neoangiogenesis by binding with VEGF [209,210,211,212]. A systematic review exhibited that when used alone or when combined with a cytotoxic drug, it prolonged the overall survival in patients with recurrent GBM by four months [209]. Bevacizumab in anaplastic astrocytoma, anaplastic oligodendroglioma, and oligodendroglioma improved overall survival, progression-free survival, and standard therapy in patients. The common toxicities are hypertension, thromboembolic events, and hypophosphatemia [213,214,215].
Depatuxizumab mafodotin (ABT-414)	Inhibits wild type *EGFR* or *EGFRVIII*, thus preventing polymerization of microtubules which is important for vesicular trafficking and mitosis of cancer cells. Modest improvement in progression-free survival among recurrent GBM patients [216,217,218].
Peptide vaccine	Peptide vaccines act against *EGFRvIII*, which is an active protein that is only expressed in GBM and not healthy tissues; rindopepimut (CDX-110) is used in clinical trials to target *EGFRvIII* in recurrent GBM patients [219,220,221,222]. Rindopepimut used in recurrent GBM patients showed a significant improvement in progression-free survival when combined with Bevacizumab [219,220]. In the ACT IV trial, whereby peptide vaccine was used for newly diagnosed GBM patients, it failed to show survival benefits when used in combination with Tmz [197].
Heat Shock Protein (HSP) vaccine	Patients treated with the HSPPC-96 vaccine in a phase-II trial showed median overall survival that is comparable to phase-I, an improvement compared to their benchmark, with or without bevacizumab (42.6 weeks vs. 14.6 months) [223,224]. HSPPC-96 demonstrated median overall survival with a high tumor-specific immune response above 40.5 months (95% CI) as compared with 14.6 months (95% CI) for patients with low tumor-specific immune response. The HSPPC-96 in combination with standard therapy, was safe in newly diagnosed GBM patients [225].
Dendritic cell (DC) vaccine	DC vaccine can immunologically present the antigens on glioma, activate CD8^+^ cells, prevent angiogenesis, and trigger tumor cell death [226,227,228]. In newly diagnosed GBM, patients treated with DCs with or without adjuvant therapy resulted in an improved median overall survival, progression-free survival, and higher survival rate of three years [189,195,229,230,231]. In Phase I/II, the use of DC-type multipeptide vaccine in patients with HGG (GBM, anaplastic astrocytoma, anaplastic oligodendroglioma, and anaplastic oligoastrocytoma) demonstrated clinical efficacy in as nine patients who were vaccinated (41%) remained free of progression for more than 12 months [197].

**Table 6:** Studies carried out that utilized DC vaccine in the treatment of HGGs. (AA—anaplastic astrocytoma, AO—anaplastic oligodendroglioma, GBM—glioblastoma, OS—overall survival, PFS—progression-free survival) [168].

Study Reference	WHO Classification of Tumor	Phase of the Clinical Trial	Total Patients		Outcome
			DC Vaccine	Placebo	
Wheeler et al. [232]	IV (GBM)	IA/IB/II	13	13	OS
Yu et al. [233]	III (AA), IV (GBM)	I	8	26	OS
Batich et al. [234]	IV (GBM)	I	11	23	OS
Der-Yang Co et al. [231]	IV (GBM)	II	18	16	OS, PFS
Chang et al. [235]	III (AA, AO), IV (GBM)	I/II	16	63	OS
Yamanaka et al. [236]	IV (GBM)	I/II	18	27	OS
Jie et al. [237]	IV (GBM)	I/II	13	12	OS
Vik-Mo et al. [238]	IV (GBM)	I/II	7	10	OS, PFS

### 4.3. Tumor-Treating Field (TTF)

Tumor-treating field (TTF) is an anti-mitotic electric field therapy that tampers with cell division and assembly of organelle via the delivery of low-intensity alternating electric field to GBM tumor [239]. Initial clinical studies in recurrent GBM patients (n = 10), shows that TTF prolonged the median time of disease progression (26.1 months), 6 months progression-free survival rates (50%) and median overall survival (>62 weeks) [240,241] TTF can enhance Tmz therapeutic efficacy by delaying the repair of damaged DNA in newly diagnosed or recurrent GBM [242,243,244,245]. TTF in combination with Tmz increases overall survival (about four months) and progression-free survival (approximately three months) with reported improvement in patients’ quality of life and low incidence of adverse effects as opposed to Tmz only (Table 7) [242,246,247]. Optune, a clinical TTF device commercialized by Novocure, has demonstrated statistically significant survival rates in recurrent GBM patients [248]. The minimally invasive nature, decreased systemic toxicity and side effects are some of the attractive properties of TTF therapy [241,248]. This is particularly important in recurrent illness, where patients undergo a variety of treatments from chemotherapy to additional surgery and/or re-irradiation [248]. Despite significant improvement with minimal adverse effects on physical and social functioning, TTF is a costly option with an average cost of 185,476 euros per patient [243,249,250,251]. Additional drawbacks include lifestyle restrictions as the device must be continuously worn due to the correlation between device compliance and overall survival [251].

## 5. Repurposing Drugs for HGG

Quinoline-based antimalarial drugs such as chloroquine and hydroxychloroquine have gained the potential to be repurposed alongside Stupp therapy. Both chloroquine and hydroxychloroquine have been studied in preclinical and clinical trials as chemo-radiosensitizer. Chloroquine promotes Tmz sensitivity by promoting apoptotic cell death while inhibiting autophagosome fusion and mitochondrial autophagy [255]. Hydroxychloroquine (5 µg/mL) synergizes Bevacizumab (100 µg/mL) inhibition of autophagy by increasing LC3-II/LC3-I ratio and p62 that causes Beclin1 degradation [256]. The formation of GSCs and the highly hypoxic HGG tumors may hinder current therapy efficacy in HGG. Chloroquine (20 nmol/L) synergistically radiosensitizes irradiation-induced apoptotic death and autophagy suppression in U87 glioma-initiating cells. The addition of chloroquine further reduced the number and diameter of glioma-initiating cells tumorsphere [257]. Chloroquine also promotes the histone deacetylation induced by histone deacetylase inhibitor, suberoylanilide hydroxamic acid, in combination with Tmz [258]. Interestingly, chloroquine cotreatment with radiation suppresses the malignancy characteristic of GBM cells by inhibiting TGF-β [259]. This inhibits matrix metalloproteinase-2, cell invasion, clonogenic formation and enhances cell death. Both chloroquine (200 mg, daily) and hydroxychloroquine (200 to 800 mg, daily) have been tested in a clinical trial to enhance the efficacy of Tmz and radiation in improving median overall survival, particularly in newly diagnosed *EGFRvIII*- and *EGFRvIII*+ GBM patients [260,261].

Repurposing older drugs such as metformin and antipsychotics is beneficial as they are relatively cheap while capable of promoting standard therapy. For instance, in response to specific Tmz concentrations, Akt activity can be activated, heightening tumorigenicity, stemness, and cancer cells’ invasiveness [262]. Hence, by down-regulating Akt activation, the cytotoxic effects of Tmz can be enhanced. Metformin showed the ability to inhibit Akt activation, thus enhancing TMZ cytotoxicity [263,264]. Additionally, antipsychotics can also be repurposed to counter the neoplastic activity of human gliomas, as reviewed extensively by Kamarudin and Parhar [265]. For instance, perphenazine, in combination with Tmz, demonstrated significant antiproliferative activity. Moreover, antipsychotic drugs such as perphenazine can cross the BBB and antagonize the dopamine receptors, namely D2 and D3, which are implicated in glioma formation [266,267].

Although the repurposing of drugs shares an adjuvant commonality in improving HGG therapy, several issues may limit their therapeutic use. Although their repurposing may offer therapeutic advantages in HGG therapy, most drugs can elicit cytotoxicity with severe side effects. For instance, most studies reported the effective concentration of hydroxychloroquine as an adjuvant to be ∼20 µM, significantly higher than its acceptable dose of ∼5 µM. Even though the current empirical evidence supports their potentiation of current therapy, it is generally accepted that such combinations would also equally enhance the side effects. Hence, it is imperative to determine the clinically acceptable range dose of these drugs, particularly in phase I/II clinical trial. Alternatively, the sequential treatment of Tmz with hydroxychloroquine and BH3 mimetic, AT101, demonstrated a higher cytotoxic effect toward GBM tumor growth but with lesser cytotoxicity in normal astrocytes as compared to treatment with Tmz alone [268]. This sequential approach may be beneficial as a clinical approach to reducing long-term treatment side effects. One of the significant problems is their capability to cross the BBB since most of these commercially available drugs have not been proven to cross the BBB.

Additionally, their bioavailability in the brain and pH stability, particularly within the HGG tumor surrounding, remains unanswered. As compared to metformin and quinolone-based antimalarial drugs, anti-psychotics agents such as selective serotonin reuptake inhibitors, tricyclic antidepressants, lithium chloride, and valproic acid are more commonly prescribed with glioma patients following the standard therapy. These antipsychotic drugs’ ability to cross the BBB further highlights their potential to be prioritized as a repurposed drug-based adjuvant in the clinical setting, as reviewed by us previously [269]. Additionally, these anti-psychotic drugs are well-studied in brain-related disorders and cancer studies. Moreover, in clinical and population-based studies, retrospective, and case-report, this group of drugs demonstrated safety profiling and promoted standard therapy. However, more conclusive data from a larger cohort and Phase III trial are still required to justify using these antipsychotic drugs towards the standard treatment.

## 6. Phytochemicals and Nanoparticles in HGG

### 6.1. Flavonoids

Flavonoids are a group of bioactive polyphenolic agents structurally diverse with low toxicity [269,270]. They have been studied for their anti-cancer properties in glioma models [271,272,273,274]. Galangin (3, 5, 7-trihydroxyflavone), a natural flavonoid from roots of *Alpinia officinarum Hance, Alnus pendula Matsum, Plantago major* L, and *Scutellaria galericulata* L. *(S.scrodifolia Fisch*.), honey, and propolis [275,276]. Interestingly, galangin is cytotoxic to tumor cells but non-cytotoxic to normal cells, making it a potential anti-neoplastic agent [276]. Galangin’s anti-cancer effects include induction of autophagy, cell cycle arrest at the G_0_/G_1_ phase, promotion of ROS-induced apoptosis, anti-angiogenesis, and anti-proliferation [275,277]. Galangin, in combination with chloroquine, suppresses tumor growth, promotes apoptosis, pyroptosis, and prolonged survival in vitro and in vivo GBM models compared to galangin monotherapy [276].

Curcumin [1,7-Bis(4-hydroxy-3-methoxyphenyl)-1,6-heptadiene-3,5-dione] has been reported with anti-proliferative, anti-angiogenesis and induction of apoptosis in numerous cancer models [20,278]. For instance, curcumin enhances inhibition of angiogenesis, cell invasion and promotes apoptosis when combined with paclitaxel in GBM cells [20,279]. Curcumin augments nimustine (ACNU) anti-tumor activity by enhancing the inhibition of P13K/Akt and NF-κB/COX-2 in GBM cells [20]. In another study, curcumin potentiated paclitaxel cytotoxicity in rat C6 glioma cells by inhibiting NF-κB activation [280]. In patient-derived GSCs, curcumin (25 µM) significantly reduces Glio 3 and Glio 9 cells’ viability via induction of ROS, activation of MAPK pathway, and downregulation of STAT3 and IAPs [281].

miRNA can play a role in treatment resistance in GBM [282,283,284,285]. Curcumin can increase miRNA expression in GBM, overcoming Tmz resistance. Li and coworkers showed that curcumin (60–120 mg/kg) induced miR-378 expression significantly inhibited tumor growth (30–60%) of xenografted U87-miR-378 in SCID mice [285]. The study also demonstrated curcumin (50 µM) significantly suppressed cell proliferation and enhanced apoptosis via p38 signaling in U87-miR-378 [285].

Although curcumin is useful in various cancers, its bioavailability and absorption are low, resulting in rapid metabolism and systemic elimination. The use of formulated nanoparticles such as poly(lactic-co-glycolic acid) [286], poly(butyl)cyanoacrylate [287], and tripalmitin-oleic acid [288] enhance curcumin distribution in vitro and in vivo models. For instance, poly(lactic-co-glycolic acid) demonstrated an increased half-life in male Sprague–Dawley rat (210 ± 10 g body weight) brain tissue from 9 to 15 min [286]. Additionally, a significant increase in curcumin retention time was reported in the hippocampus (83%) and cerebral cortex (96%). Curcumin loaded in tripalmitin-oleic acid [288] showed an IC_50_ reduction (80 µg/mL to 20 µg/mL) in A172 cells and further reduced tumor volume (82%) in subcutaneous flank tumor-bearing female nude mice.

Quercetin (3,3′,4′,5,7-pentahydroxyflavone) can promote apoptosis and cell cycle arrestment at G_1_ phase via cyclin-dependent kinase (CDK)-4 and cyclin D1 through p53 activation [274,289]. Additionally, quercetin targets the P13K/Akt/mTOR, IL-6/STAT, and apoptotic protein modulation [274,290,291,292]. Quercetin (30 µmol/L) in combination with Tmz (100–200 µmol/L) promoted Tmz-induced growth inhibition in U87 and U251 via Hsp27 inhibition [293]. Quercetin combination with chloroquine (CQ) induced caspase-dependent apoptosis, autophagic inhibition, and lysosomal suppression in T98G cells [294]. Additionally, quercetin (50 µM) and CQ (20 µM) induced ER stress in T98G cells. In another study, quercetin (25 µM) in combination with sodium butyrate (1 mM) induced apoptosis in rat C6 and T98G cells by modulating Bax, Bcl-2, and survivin proteins that led to caspase-3 activation and PARP [295].

Resveratrol (3,4′,5-trihydroxy-trans-stilbene) anti-cancer effects include anti-proliferation, cell cycle arrestment, and apoptosis promotion through multiple signaling pathways such as EGFR, p53, P13K/AKT/mTOR, STAT3, NF-κB, and oncogenic miRNAs [296,297]. Resveratrol suppresses tumor growth and prolongs survival in rats bearing intracranial C6 glioma [296,298,299]. Wang and coworkers showed a longer mean survival in C6-xenograft rats treated with resveratrol (29.75 ± 9.27 days) than the control group (15.8 ± 0.93 days) [296]. Resveratrol administration decreased the expression of EGFR, MMP-9 NF-κB, PCNA, COX-2, and VEGF while increasing GFAP expression compared to the control group. Resveratrol also enhances Tmz efficacy by reducing ROS/ERK-mediated autophagy and promoting apoptosis [297,299,300]. Resveratrol in combination with Tmz suppresses the cell growth and induces apoptosis in RG-2 cell (>20%, 17%), LN-18 (62.3%, 12%), and LN-428 cells (28.6%, 8%) [297]. The co-treatment also reduce MGMT protein expression RG-2 (44.9%), LN-18 (38.7%), and LN-428 (33.5%) compared to the Tmz-treated group only. Additionally, resveratrol administration via lumbar puncture effectively suppresses intracranial tumor growth in orthotopic rats and prolonged survival [299,301,302]. Combination therapy with neurosurgery and lumbar-punctured resveratrol demonstrated significant improvement of survival post-operation in orthotopic rats by inhibiting tumor growth, promoting apoptosis, and inactivation of STAT3 [299,303]. The use of resveratrol-loaded polyethylene glycol-polylactic acid nanoparticles with transferrin moieties (Tf-NP-RES) reduced tumor volume and prolonged the survival in C6 orthotopic rats and U87MG-xenograft mice [304,305]. The use of liposomal TriCurin (TrLp; curcumin: epicatechin gallate: RES 4:1:12.5) synergistically enhanced resveratrol anticancer effects through the upregulation of p53 proteins in GL261 cells and C57BL/6 male mice implanted with GL261 cells [306].

### 6.2. Polysaccharides

Polysaccharides possess immunomodulatory properties and are often referred to as “biological response modifiers” [304,305], contributing to their therapeutic value as anticancer agents. Polysaccharides modulate transcription factors and transcription of genes associated with cell proliferation, angiogenesis, metastasis, cell cycle arrest, and apoptotic induction [307,308,309]. Schizophyllan is a (1→3)-β-D-glucan rich polysaccharide found in the fungus *Schizophyllum commune.* Zhou and coworkers showed that schizophyllan reduced tumor growth in a dose-dependent manner in male Sprague Dawley rat models implanted with the intracranial tumor in situ (20 mg/kg: 30.8 ± 4.1%, 40 kg/mg: 38.3 ± 3.5%, 60 mg/kg: 55.3 ± 5.1%) compared to control group [310]. In vitro study on CNS-1 rat glioma treated with 40 and 60 mg/L schizophyllan showed a reduction in cell number, increased apoptosis, and cell cycle arrestment at G_0_/G_1_ phase [310]. Fucoidan is a sulfated polysaccharide, commonly found in brown algae (*Laminaria digitata, Ascophyllum nodosum*, and *Fucus vesiculosus*) and brown seaweeds [249,311]. Its bioactivities include anti-tumor, immunoregulatory, and anti-inflammatory effects [311,312]. Oligo-fucoidan, a glycolytic cleavage product fucoidan (brown seaweed, *Laminaria japonica)* inhibits the cell proliferation of U87MG and GBM8401 cells compared to SVGp12 cells [249]. The study also demonstrated oligo-fucoidan ability to inhibit the expression of DNA methyltransferases 1, 3A, and 3B, induce differentiation of cell markers (MBP, OLIG2, S100, GFAP, NeuN, and MAP2), and decrease methylation of p21 (DNMT3B target gene). Additionally, the addition of decitabine (DNMT inhibitor) to oligo-fucoidan promoted inhibition of U87MG cell growth and induced myelin basic protein [249].

*Ganoderma lucidum*, commonly known as “Reishi” in Japan and “Lingzhi” in China, is a mushroom used in Asian countries for its medicinal values [313,314]. *G. lucidum* polysaccharides (GL-PS) are the bioactive component of the fungus, which possess immunomodulatory and anticancer properties [315]. GL-PS inhibited U251 cell proliferation by blocking cell cycle at G_0_/G_1_ and promoted apoptosis via caspase-3 activation [316]. In a separate study, the authors demonstrated an increase in the concentration of IL-2, TNF-α, and IFN-γ following GL-PS administration in Male Fischer rats (F344) bearing RG2 glioma [314]. The abdominal injection promoted functional maturation of dendritic cells leading to inhibition in tumor growth (101.93 ± 53.58, 113.56 ± 39.76, 161.28 ± 56.69 mm^3^) and increased median survival (27.67 ± 2.87, 31.78 ± 6.38, 27.33 ± 4.97 days) compared to control rats (162.99 ± 48.34 mm^3^, 24.44 ± 2.55 days) [314].

Lentinan (*Lentinus edodes,* also known as the shiitake mushroom), is an attractive polysaccharide with reported minimal toxicity and pharmacological properties, including antitumor, immunomodulatory, antioxidant, and blood lipid reduction [317]. Lentinan elicits its immunomodulatory properties by activating macrophages and dendritic cells via Dectin-1 receptor binding, resulting in the elevation of cytotoxic T lymphocytes and natural killer (NK) cells [318,319]. Lentinan as a monotherapy or in combination with chemotherapy has been extensively studied in osteosarcoma [320], breast [321], and ovarian cancer [322]. However, to date, lentinan has only been studied on C6 glioma cells, demonstrating anti-proliferative, cell cycle arrestment at G_0_/G_1_ phase and apoptosis induction [323]. Such findings propose lentinan as a potential phytochemical that should be explored more in preclinical HGG models.

### 6.3. Cannabinoids

Cannabinoids from *Cannabis* possess anti-cancer properties and are primarily used in cancer patients as part of palliative care to relieve pain, relieve nausea, and stimulate appetite [324,325]. Nabiximols trademarked as Sativex^®^, which contains equal parts Δ^9^-Tetrahydrocannabinol (THC) and cannabidiol (CBD) (1:1) is formulated as an oro-mucosal spray that allows slow absorption through the mucus, with rapid and direct access to the circulation, where plasma concentration plateaus more rapidly [326,327,328]. The combination of phytocannabinoids inhibited tumor growth via anti-angiogenesis and induction of apoptosis [326,327,328] in vitro (U87 and T98G) and orthotopic glioma murine models [329,330]. The phytocannabinoids combinations (THC and CBD (1:1 ratio)), when co-administered with Tmz, demonstrated strong synergistic reduction of glioma initiating cell growth in orthotopic xenograft nude mice [331]. Sativex has been explored in a clinical setting combined with Tmz (NCT01812603) in placebo-controlled phase II clinical trials involving recurrent GBM patients [328]. In a study conducted by GW Pharmaceuticals, GBM patients with 60% or greater Karnofsky performance who received dose-intense Tmz (100 µL of solution containing 27 mg/mL THC and 25 mg/mL CBD (12 sprays) reported a one-year survival rate of 83% and a median survival over 662 days compared to control group (44% and 369 days) who received Tmz only [328,332].

### 6.4. Thymoquinone

Thymoquinone (2-methyl-5-isopropyl-1, 4-benzoquinone) from *Nigella sativa* (black seed) [333] possesses anti-angiogenesis, anti-invasion, and anti-metastasis in various cancers with minimal effect on normal cells [334,335,336]. Thymoquinone also enhances the efficacy of chemotherapeutic drugs when used in combination in cancer models [337]. Thymoquinone (3.6 µM) addition to chloroquine (4.4 µM) suppresses autophagic flux, inhibits cell proliferation in T98G and Gli36ΔEGFR cells independent of the p53 status [338]. Thymoquinone (50 µM) synergized Tmz (100 µM) effects by enhancing the inhibition of U87MG cell migration and invasion, significantly more significant than Tmz or thymoquinone alone [334]. However, thymoquinones’ lipophilicity hinders its pharmacokinetics resulting in low membrane permeability, solubility, and bioavailability [333,337].

### 6.5. Potential and Challenges of Phytochemicals and Nanoparticles

**Figure 3:**
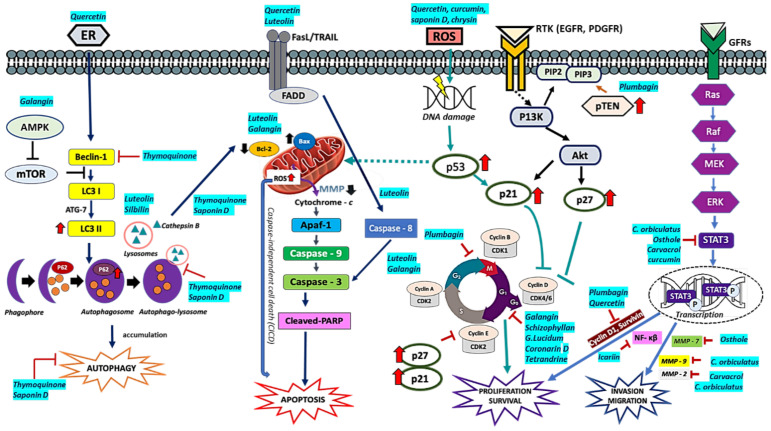
Phytochemicals as potential adjuvants in HGG. Phytochemicals from different classes modulate various signaling pathways in human HGG tumor cells that promote cell cycle arrestment, inhibit cell proliferation, invasion, migration, and promote cell death.

The discovery of plant-derived bioactive compounds as novel therapeutics may provide therapeutic advantages in HGG research (Figure 3, Table 8). Around 60 percent of commercially available clinically approved anti-cancer medications are derived from medicinal plants [339,340]. Their multitarget, high selectivity against cancer cells, capable of reducing multidrug chemoresistance, inexpensive and marginal side effects make them valuable potential therapeutics, especially when combined with current therapy advancement [341]. Phytochemicals such as thymoquinone, cannabinoids, and resveratrol have proven to enhance the anti-cancer effect in pre-clinical models when combined with Tmz [297,328,334]. Such development in pre-clinical findings further necessitates clinical studies to fully assess phytochemicals efficacy in combination with current standard therapy. Although Sativex (THC:CBD, 1:1) clinical trial (NCT01812603) demonstrated an increase in 1-year survival rates in combination with Tmz (83%) over standard therapy with Tmz alone (44%), the clinical trial did not progress any more than phase II. Hence further clinical inspection should be considered for further validation [328]. Although various phytochemicals demonstrated pre-clinical potential, their use in the animal or actual clinical setting is still not convincing and well-studied. Thus, incorporating these phytochemicals with nanoparticles delivery systems may be of interest to researchers.

Cancer nanomedicine has emerged as a revolutionary approach in cancer research, changing cancer therapeutics’ paradigm [342]. The recent rapid development of nanomaterials brings an exciting opportunity to deliver various therapeutics to sites of interest in patients while preserving healthy tissues and organs [343]. This therapeutic approach has also been approved in the ovarian and breast cancer model by the United States Food and Drug Administration and the European Medical Agency due to lesser side effects and better safety profile than the conventional therapeutic [343]. Nanomaterials such as liposomes, nanoemulsion, polymeric micelles, and iron oxide nanoparticles have been investigated as therapeutics carriers to treat HGG (Table 6). These materials demonstrated a favorable effect, enhanced permeability, and retention through positive targeting that allows nanomaterials to retain tumor tissues. Nanoparticles can cross the BBB and maintain in GBM tissue due to the “leaky” BBB caused by necrosis and microvascular proliferation of GBM cells [344]. Such properties have enabled nanoparticles to be explored in clinical settings. In early-phase clinical trials, liposome-based nanomedicines using single-agent therapy of nanoliposomes containing doxorubicin (NCT02766699) and irinotecan are currently in development (NCT02022644) [345,346]. The current status of these clinical trials is still ongoing with active recruitment.

The use of nanoparticles also faces challenges such as immune response, blood flow, red blood cells hemolysis, and substantial tissue resistance, preventing nanoparticles from being internalized cellularly, particularly in the nano-drug diffusion in vivo model [347]. In phase II clinical trial, postoperative GBM patients who underwent chemoradiation did not show statistically significant benefit in the overall survival and 6-month progression-free survival when subjected to the combination of Tmz and pegylated liposomal doxorubicin [348]. Similarly, intraperitoneal administration of pegylated liposomal Tmz in glioma bearing male Lewis rats and in vitro study (CNS-1 glioma cancer cells) demonstrated prolonged survival and decreased tumor volume. However, such effects were not statistically significant [349]. These unsuccessful events could be due to the mode of delivery, non-specific, non-targeted, and reduced drug availability impeded by the BBB and tumor heterogeneity. Therefore, a more precise and target-specific nano therapy is required. The use of doxorubicin as a standard drug in this in vitro study demonstrated ITGα-2 expression in GBM to be significantly higher than EGFR [350]. Doxorubicin delivered by GBM-induced angiogenesis selectively via ITGα-2 antibody-directed liposome improved anti-tumor efficacy and penetrated BBB (cells A172 and U87), which highlights ITGα-2 as a potential strategy.

**Table 8:** Pre-clinical and clinical studies on the use of natural products in GBM treatment.

Phytochemical	Study Design	Observations
Curcumin	U118, U87, U251MG-100 µM nimustine hydrochloride + 20 µM curcumin	Enhanced anti-proliferation, anti-migration, and proapoptotic activities of nimustine hydrochloride [20].
	Patient-derived GSCs (Glio 3, Glio 9)—25 µM curcumin	Reduced cell viability of GSCs via ROS-dependent mechanism, MAPK-pathway activation and downregulation of STAT3 and IAPs [281].
	U87-miR-378-50 µM c SCID mice-30, 60, 120 mg/kg	miR-378 sensitized GBM toward curcumin, inhibited tumor growth, cell proliferation, and induce apoptosis [285].
Thymoquinone	U87MG-50 µM TQ + 100 µM Tmz	Decreased cell migration and invasion [334].
Plumbagin	A172, U251-5.5 μM (IC_50_)	Cell cycle arrestment at G_2_/M phase. Apoptotic induction with minimal necrotic cell death. PTEN overexpression and downregulation of E2F1, MDM2, cyclin B1, surviving, Bcl-2 protein, and PARP-1. Inhibition of telomerase activity [351].
Sativex	NCT01812603 Phase I and Phase II (n = 21 GBM) with Karnofsky performance scale ≥60% 100 µL (12 spray/day) Sativex (27 mg/mL THC + 25 mg/mL CBD) orally + Tmz Control: Tmz alone	83% of one year survival rate in Sativex + Tmz group compared to 44% in Tmz alone [328].
Quercetin	T98G-50 µM quercetin + 20 µM chloroquine	Induced autophagy and ER stress [294].
	C6, T98G-25 µM quercetin + 1mM NaB	Promoted apoptosis via increased expression of Bax, caspase 3, downregulation of Bcl-2, surviving and PARP degradation [295].
Resveratrol	C6-50,100,150 µM	Inhibited cell proliferation, cell cycle arrestment at s-phase, apoptotic induction, downregulation of miR-21, miR-19 and miR30a-5p [296].
	RG-2-25 µM Resveratrol + 250 µM Tmz LN18, LN428-75 µM Resveratrol + 750 µM Tmz	Inhibition of MGMT expression, downregulation of STAT3/Bcl-2/surviving, apoptosis and cell cycle arrestment (G1 or S-phase) [297].
Galangin	U87MG and U251-100 µM	Apoptosis, cell cycle arrest G_0_/G_1_ pytoptosis, and protective autophagy. Enhanced chloroquine-suppressed tumor growth compared to galangin monotherapy [276].
	Male BALB/c athymic mice, 4 weeks old; 14–17 g) (orthotopic U87MG xenograft) 100 mg/kg/day GG + 25 mg/kg/day chloroquine; control: DMSO	
Schizophyllan	CNS-1-40 and 60 mg/L Schizophyllan	Apoptosis and cell cycle arrest at G_0_/G_1_ phase. Tumor growth inhibited [310].
	Sprague Dawley male rats (n = 40) (in situ intracranial tumors, CNS-1) 20, 40, 60 mg/kg; control 0.9% NaCl	
Icariin	U87MG-10 µM ICA + 200 µM Tmz	Synergistically decreased cell proliferation, sensitized GBM cell by enhanced apoptosis by increased caspase-3 and cleaved PARP expression. Inhibited cell migration, invasion via suppression of NF-κB activity [352].
Silbinin (*Silybum*)	A172, SR-50, 100, 150 µM s	Apoptotic induction via caspase-3 activation and PARP-1 cleavage. Enhanced autophagic flux via LC3-I to LC3-II conversion and P62 degradation. Inhibition of mTOR and downregulation of YAP [353].
Luteolin	U251, LN229-10, 20 30 µM	Inhibited cell proliferation. Apoptotic induction via MAPK by activation of FADD, upregulation of cleaved PARP, cleaved caspase-8, and cleaved caspase-3. Increased expression of Bax to Bcl_2_ ratio. Autophagy induction promoting miR-124-3p expression [354].
Silbinin + Luteolin	U87, T98G-50 µM SIL + 20 µM	Synergistically inhibited cell proliferation, invasion, and migration. Apoptosis induction and inhibition of rapamycin (RAPA)-induced autophagy via iNOS downregulation, PKCα suppression, and miR-7-1-3p upregulation [355].
	Female nude mice (nu/nu) (subcutaneous U87MG, T98G xenografts) Silbinin (200 mg/kg/day) + Luteolin (10 mg/kg/day)	
Oligo-fucoidan	GBM8401, U87MG-50, 100, 200 µg/mL	Cell cycle arresting at G_1_/S phase induced cell differentiation, inhibited DNA Methyltransferases, and decreased p21 methylation [249].
*G. lucidum* polysaccharides (GL-PS)	U251- 50, 100, 200, 400 or 800 μg/mL	Inhibited cell proliferation, cell cycle arrestment at G_0_/G_1_ phase, promote apoptosis via caspase 3 activation. Increased IL-2, TNF-α, INF-γ. Enhanced cytotoxicity of NK and T cells. Inhibited tumor growth and prolonged rat survival [316].
	Male Fischer rats (200-250G)- 50, 100, and 200 mg/(kg d) GL-PS; control: saline	
Saponin D (*Pulsatilla koreana*)	U87 MG-10 μM SB365	Inhibited cell proliferation. Alteration in mitochondrial membrane potential (MMP), neutralization of lysosomal pH Increased ratio of LC3-II/I and p26 in cell indicating Inhibition of autophagic influx mediated by cathepsin B and mainly ROS. Co-treatment of SB365 and Tmz exerted an additive effect. Suppression of tumor growth in xenograft model [356].
	Nude mice-SB365 (5 mg/kg/every other day, intratumoral) + Tmz (2.5 mg/kg/day, i.p., U87 xenograft)	
Toosendanin	U87, C6, T98G-10 nM	Inhibited cell proliferation and induced apoptosis in vitro and in vivo. Reduce tumor progression via apoptosis. Reduced tumor weight. Increased expression of Bax, cleaved caspase-3, and reduction in Bcl-2 expression. No cytotoxic effect in T98G. Apoptosis induced via increased expression of estrogen receptor *β* and p53 [357].
	Athymic nude mice—6 weeks old (n = 10), (U87-Luc xenograft, subcutaneous) 1 mg/kg qd (orally)	
Coronarin D	U251-10, 20, 40 μM	Cell cycle arrest at G_1_ phase, induced caspase-dependent mitochondrial-mediated apoptosis by increasing phosphorylated ERK, p-H2AX histone, and overexpression of p21 [358].
Carvacrol	U87-500 μM	Inhibition of TRPM7. Reduction in cell viability, migration, invasion, and MMP-2. Promotion of cofilin phosphorylation and inhibition of Ras/MEK/MAPK and PI3K/Akt. TRPM7 [359].
Lentinan	C6- 20, 40, 80 mg/L	Inhibited tumor growth, cell proliferation, cell cycle arrestment at G_0_/G_1_ phase, and promoted apoptosis [323].
	SD male rats-20, 40, 80 mg/kg/d; control: 0.9% Nacl	
*Ficus carica*	U138 MG, T98G, U87 MG-0.25 mg/mL	Inhibited GBM cell proliferation, and stimulated apoptosis. Inhibit cell invasion via reduction in VEGF expression. Synergistic inhibition in GBM cell proliferation. The co-treatment increased miRNA expression (let-7d) in T98G cells modulating GBM progression via miRNA [360].
	U138 MG, T98G-0.25 mg/mL + 450 μM Tmz U87 MG-0.25 mg/mL + 25μM Tmz	
*Celastrus orbiculatus*	U87, U251-20, 40, 80 μg/mL	Inhibition of cell adhesion, migration, and invasion. Reduction in N-cadherin, vimentin, MMP-2, and MMP-9 expression. Upregulation of E-cadherin. Inhibition in actin assembly. [361].
Tetrandrine (*Stephania tetrandra*)	U87, U251-4 μM Tet + 2 Gy	Enhanced radiosensitivity of the cell. Inhibited cell proliferation by decreasing phosphorylated ERK expression. Cell cycle arrestment at G_0_/G_1_ phase [362].
Osthole	U87-50, 100, 200 μM	Inhibited cell proliferation and enhanced apoptosis in cells. Increased expression of miR-16 precursor and decreased expression of MMP-9 [363].
Trichosanthin	U87, U251-10, 20 μM	Inhibited cell proliferation, invasion and migration. Induced apoptosis and inhibited LGR5 expression suggesting repression in Wnt/*β* - catenin signaling pathway [364].

## 7. Precision Medicine

Precision medicine is a type of customized treatment that can be used to treat patients with HGG according to their specific molecular profile [365,366]. One example is using the novel 3D brain cancer chip, which utilizes GBM cells to form 3D cancer tissues for drug screening, therapy resistance, and tumor cell motility [367,368,369,370]. For instance, the use of poly(ethylene glycol) diacrylate (PEGDA) hydrogel, thereby making it permeable to biomolecules and water, allows “smart release” of the chemical transported on the chip to study the response of the drug in the adjacent 3D environment [367]. Utilizing the concept of PEGDA hydrogel, this can be applied in the polyvalent vaccine, which may confer better advantages than monovalent vaccines. However, its large molecular size may pose a challenge to cross the BBB. Hence, by integrating PEGDA hydrogel in it, this challenge could be overcome. The ability of induced neural stem cells (iNSCs) derived from patients’ skin cells to cross the BBB makes it an ideal candidate to be used for personalized therapy in GBM treatment [22]. iNSCs are genetically engineered to have the ability to undergo differentiation while triggering apoptosis in co-cultured human GBM cells [22,196]. In a study by Bago et al. in 2016, the authors proved that the delivery of TNF-α-related apoptosis-inducing ligand (TRAIL) via iNSCs in murine GBM models resulted in a decreased growth of diffused and solid GBM xenografts by 20 and 230-fold respectively.

Additionally, it also prolonged the median survival in these murine models [371]. The data support the potential of iNSC being a highly efficient drug-delivery vehicle for the treatment of both invasive and solid brain tumors [371] Hence, more preclinical studies are required to determine the efficacy and potentiality of iNSCs before considering it in GBM treatment. Moreover, molecular genetic tools would help to determine a patient’s prognosis and the best therapeutic regimen for each patient. For instance, patients with triple-positive mutations (1p/19q codeletion, *IDH* mutation, and *TERT* promoter mutation) have a favorable prognosis, while patients with triple-negative mutation often have poorer prognoses [372,373,374,375]. This information can be used to ensure the patients whose prognosis is favorable are not treated too aggressively at the onset of the disease to prevent treatment-induced neurological deficits. Hence, in precision medicine, a prognostic marker can be determined, which could be used to plan the treatment mode, eventually improving the patients’ prognoses.

In an article by Prados et al. [376], to illustrate the principles of molecular profiling of GBM, the authors carried out genome and exome-wide sequencing of 13 samples of recurrent GBM. They mapped the identified genomic alterations to possible CNS-active treatment modalities. One of the recurrent GBM samples exhibited *CDKN2A* gene deletion, *EGFR* gene amplification, and *EGFRvIII* expression [376]. The therapeutic agents which served as strong candidates for GBM with amplification of the *EGFR* gene include afatinib, dacomitinib, and propranolol. Afatinib is an irreversible EGFR/ERBB2 inhibitor [376]. In preclinical trials, it has been shown to have activity against the *EGFRvIII* variant. However, afatinib’s efficacy in GBM is not demonstrated yet [376]. Dacomitinib is also an EGFR inhibitor and is currently tested in GBM clinical trials (NCT01112527). It is reported that dacomitinib have improved penetration of the BBB [376]. Propranolol, commonly used in hypertension, migraine prophylaxis, angina pectoris, and various other conditions, has recently exhibited the ability to control EGFR trafficking. However, its efficacy in clinical trials remains to be seen [376]. For *CDKN2A* deletion, the therapeutic agent of choice includes cyclin-dependent kinase (CDK) 4/6 inhibitors. One example is PD-0332991, which is currently in phase II of GBM clinical trials (NCT01227434), as mentioned in an article by Prados et al. [376]. Using this GBM sample as an example, if an EGFR inhibitor that has activity against *EGFRvIII* and can penetrate the BBB is coupled with a CDK 4/6 inhibitor, it may serve as a potentially effective treatment strategy in this case. Another recurrent GBM sample exhibited mutation of *BRAF* V600E gene, deletion of *TSC2, FANCA* and *RECQL5* genes [376]. These deletions and mutation can cause the activation of both the MAPK and P13K/mTOR signaling pathways. In this context, if an mTOR inhibitor coupled with a BRAF/MEK pathway inhibitor is utilized, it could be a potentially effective treatment mode in this case [376]. These two examples exhibit the importance of precision medicine in HGG.

One way precision medicine could be applied is by acquiring multiple biopsies of the tumor mass during surgery, which includes both the enhancing and the non-enhancing regions of the particular HGG [376]. Then, extensive profiling of the genome is performed, and the drugs which are considered the most probable candidates to serve as the therapeutic agent of choice are selected. All the drug selections can be individualized to tackle the various genetic alteration of the HGG. Additionally, some samples of the tumor are also collected for future xenograft testing. Blood samples are also acquired over time so that tumor DNA that is circulating can be assessed. This may help for the future development of non-invasive biomarkers [376]. In short, precision medicine will help to combat the heterogeneity and complex nature of GBM strategically.

## 8. Conclusions

The introduction of newer therapies like immunotherapy or gene therapy has provided some improvement in HGG patients. However, prolongation of overall survival does not translate into the eventual prospect of curing this disease. Immunotherapy, although promising, is yet to demonstrate anti-tumor efficacy in human HGG. This may be due to the complex immune mechanisms and tumor heterogeneity that have not been fully understood. These approaches should be pursued, perhaps by trying to reactivate the tumor-immune system several times until the tumor has completely disappeared. The different subtypes of GBM (neural, proneural, mesenchymal, and classical) have made the disease even more complicated. Thus, how each different subtype responds with the other immunotherapies remains unclear. Another challenge is to ensure that immunotherapy and chemoradiation are used strategically when used in combination. The side effects of chemotherapy and radiotherapy may pose an obstacle to immunotherapy efficacy; thus, timing is crucial when used in combination.

Although fascinating, the current therapeutic approaches, such as immunotherapy, are accompanied by many drawbacks such as time-consuming, materials used, and complexity of the experimental design. Therefore, a more cost-friendly with high specificity towards tumors with marginal side effects such as the use of phytochemicals and the repurposing of older drugs should be further considered in HGG treatment. Moreover, repurposing older drugs with the innovations mentioned above provides a multitarget molecular approach while being cost-effective in HGG management. Although these phytochemicals and older drugs’ bioavailability is a major problem, formulation and combination therapy have shown as a solution to address such issues. Studies focusing on the use of novel nanoformulations to improve the bioavailability and efficacy of flavonoids and other lipophilic compounds are vital. Moreover, the co-administration of phytochemicals, immunotherapy, and older drugs with standard chemotherapeutic drugs mainly results in modulating multiple signaling pathways. Thus, the use of nano targeted delivery may provide a clinical perspective in HGG therapy.

Hence, precision medicine with the integration of the discussed therapeutic advancements may be the future trend to find a cure via extensive genetic profiling. In short, a multimodal approach is required to treat HGG as no single method is considered adequate, with surgical resection being an integral part of this approach. More importantly, the current established use of chemotherapy, surgical resection, and radiotherapy do not guarantee a complete remission or tumor resection in HGG patients. Therefore, the combination of various therapeutic approaches may provide a better alternative to exclusively treat and target HGG tumor with different subtypes while delivering a safer toxicity profile in patients with HGG.

## Figures and Tables

**Table 1:** Innovations in neurosurgical oncology.

Innovation	Description
Awake Craniotomy	Allows identification of eloquent areas of tumor in the subcortical and cortical regions, especially tumors which would otherwise be regarded as inoperable [24,26]. Allows monitoring of patient while awake during surgery, thus increasing the degree of resection. Better Karnofsky Performance Score post-operatively, local anesthesia usage, and decrease hospitalization [24,27,28]. Patients generally had better resections than patients under general anesthesia (25.9% vs. 6.5%) [24,27].
5-Aminolevulinic acid (5-ALA)	Used in fluorescence-guided surgery, allowing to determine the tumor location, investigate MRI findings pre and post-operatively, and identify the eloquent areas involved in surgery [29,30]. Exhibits promising results in increasing the patient’s survival with gross total resection more achievable than without 5-ALA (65% vs. 35%, respectively) [24,29,30,31]. Adverse effects—increased liver enzymes, neurological impairment and photosensitivity [24,32].
Intraoperative mass spectrometry (MS) and Desorption electrospray ionization (DESI)	Used to determine how molecules are arranged spatially in biological tissues [24]. Integration of MS allows surgeons to distinguish tumors by acquiring the complex molecular data in real-time [33,34,35]. DESI allows biological tissues to be directly sampled and analysis of molecules that are intact [34,36,37]. 83% and 93% value for specificity and sensitivity respectively of surgical demarcation when estimating the percentage of high tumor cell using DESI-MS [33].
Carmustine (BCNU) wafers (Gliadel^®^)	During surgery, carmustine (BCNU) is implanted at the tumor site. This enables carmustine (BCNU) to diffuse across the adjacent tissues and supply therapeutic doses locally [38,39]. The combination of Gliadel wafers with systemic Tmz and radiotherapy prolonged the overall survival [38,39].

**Table 7:** Tumor-treating field (TTF) and adjuvants in HGG.

	Study Design	Treatment Intervention	Outcomes
Dendritic cell (DC) vaccine	Phase II—randomized, double-blind, controlled study (n = 124 newly diagnosed GBM without chemoradiation) NCT01280552	Patients ratio, 2:1 ICT-7 (n = 81) Placebo DC (n = 43)	18.3 months overall survival for ICT-7 group vs. 16.7 months control group [252].
	Phase III—a randomized trial (n = 331 GBM post-surgery and chemoradiation) NCT00045968	Patients ratio, 2:1 Tmz + DCVax-L (n = 232) Tmz + placebo (n = 99)	Median overall survival of methylated *MGMT*—34.7 months, with 3 years OS (46.4%) [189].
Tumor-treating fields (TTF)	Phase III—randomized, open label-trial (n = 695 GBM with resected tumors and completed chemoradiation)	Patients ratio, 2:1 Tmz + TTFields (n = 466) Control: Tmz alone (n = 229) TTF—18 h/day followed with Tmz (150–200 mg/m^2^/day) for 5 days (28 cycles).	TTF with chemoradiation increased overall survival from 16 months (Tmz alone) to 20.9 months [242].
Nanoparticles	In vitro SF-763, and U-118MG cell lines	Iron Oxide Nanoparticle conjugated with Cyclodextrin and Chlorotoxin and loaded with fluorescein and paclitaxel	Selectively targeted GBM cell line, effectively killing *MGMT*-resistant GBM cells [253].
	In vivo Wild type mice IV administration	Gemcitabine + Chlorotoxin Conjugated Iron Oxide Nanoparticle + Hyaluronic acid	Increased half-life (blood) 2.8 h, 10-folds higher than free GEM mice [254].

## Abbreviations

DESI—Desorption electrospray ionization; *EGFRvIII*—Epidermal growth factor receptor variant III; GBM—Glioblastoma; GSC—Glioma stem cell; HGG—High-grade glioma; LGG—Low-grade glioma; *MGMT*—O6-methylguanine–DNA methyltransferase.

## References

[1] Bray F., Ferlay J., Soerjomataram I., Siegel R.L., Torre L.A., Jemal A. (2018). Global cancer statistics 2018: GLOBOCAN estimates of incidence and mortality worldwide for 36 cancers in 185 countries. Cancer J. Clin..

[2] Siegel R.L., Miller K.D., Jemal A. (2015). Cancer statistics, 2015. Cancer J. Clin..

[3] Siegel R.L., Miller K.D., Jemal A. (2020). Cancer statistics, 2020. Cancer J. Clin..

[4] Tamimi A.F., Juweid M. (2017). Epidemiology and outcome of glioblastoma. Exon Publ..

[5] Diwanji T.P., Engelman A., Snider J.W., Mohindra P. (2017). Epidemiology, diagnosis, and optimal management of glioma in adolescents and young adults. Adolesc. Health Med. Ther..

[6] Louis D.N., Perry A., Reifenberger G., Von Deimling A., Figarella-Branger D., Cavenee W.K., Ohgaki H., Wiestler O.D., Kleihues P., Ellison D.W. (2016). The 2016 World Health Organization classification of tumors of the central nervous system: A summary. Acta Neuropathol..

[7] Cahill D., Turcan S. (2018). Origin of gliomas. Seminars in Neurology.

[8] Hervey-Jumper S.L., Berger M.S. (2016). Maximizing safe resection of low-and high-grade glioma. J. Neuro Oncol..

[9] Ostrom Q.T., Gittleman H., Xu J., Kromer C., Wolinsky Y., Kruchko C., Barnholtz-Sloan J.S. (2016). CBTRUS statistical report: Primary brain and other central nervous system tumors diagnosed in the United States in 2009–2013. Neuro Oncol..

[10] Fangusaro J., Bandopadhayay P. (2020). The “Risk” in Pediatric Low-Grade Glioma. Cancer Cell.

[11] De Blank P., Bandopadhayay P., Haas-Kogan D., Fouladi M., Fangusaro J. (2019). Management of pediatric low-grade glioma. Curr. Opin. Pediatr..

[12] Barnholtz-Sloan J.S., Ostrom Q.T., Cote D. (2018). Epidemiology of brain tumors. Neurol. Clin..

[13] Pretanvil J.-A., Salinas I.Q., Piccioni D.E. (2017). Glioblastoma in the elderly: Treatment patterns and survival. CNS Oncol..

[14] Bauchet L., Ostrom Q.T. (2019). Epidemiology and molecular epidemiology. Neurosurg. Clin..

[15] Taylor O.G., Brzozowski J.S., Skelding K.A. (2019). Glioblastoma multiforme: An overview of emerging therapeutic targets. Front. Oncol..

[16] Marra J.S., Mendes G.P., Yoshinari G.H., da Silva Guimarães F., Mazin S.C., de Oliveira H.F. (2019). Survival after radiation therapy for high-grade glioma. Rep. Pract. Oncol. Radiother..

[17] Khan M., Sharma A., Pitz M., Loewen S., Quon H., Poulin A., Essig M. (2016). High-grade glioma management and response assessment—recent advances and current challenges. Curr. Oncol..

[18] Harder B., Blomquist M., Wang J.W., Kim A., Woodworth G., Winkles J., Loftus J., Tran N. (2018). Developments in Blood-Brain Barrier Penetrance and Drug Repurposing for Improved Treatment of Glioblastoma. Front. Oncol..

[19] Vengoji R., Macha M.A., Batra S.K., Shonka N.A. (2018). Natural products: A hope for glioblastoma patients. Oncotarget.

[20] Zhao J., Zhu J., Lv X., Xing J., Liu S., Chen C., Xu Y. (2017). Curcumin potentiates the potent antitumor activity of ACNU against glioblastoma by suppressing the PI3K/AKT and NF-KB/COX-2 signaling pathways. OncoTargets Ther..

[21] Abbas M., Kausar S., Cui H. (2020). Therapeutic potential of natural products in glioblastoma treatment: Targeting key glioblastoma signaling pathways and epigenetic alterations. Clin. Transl. Oncol..

[22] Jain K.K. (2018). A critical overview of targeted therapies for glioblastoma. Front. Oncol..

[23] Opoku-Darko M., Amuah J.E., Kelly J.J.P. (2018). Surgical Resection of Anterior and Posterior Butterfly Glioblastoma. World Neurosurg..

[24] Lara-Velazquez M., Al-Kharboosh R., Jeanneret S., Vazquez-Ramos C., Mahato D., Tavanaiepour D., Rahmathulla G., Quinones-Hinojosa A. (2017). Advances in brain tumor surgery for glioblastoma in adults. Brain Sci..

[25] Palmer J.D., Siglin J., Yamoah K., Dan T., Champ C.E., Bar-Ad V., Werner-Wasik M., Evans J.J., Kim L., Glass J. (2015). Re-resection for recurrent high-grade glioma in the setting of re-irradiation: More is not always better. J. Neuro Oncol..

[26] Eseonu C.I., Rincon-Torroella J., ReFaey K., Lee Y.M., Nangiana J., Vivas-Buitrago T., Quiñones-Hinojosa A. (2017). Awake craniotomy vs craniotomy under general anesthesia for perirolandic gliomas: Evaluating perioperative complications and extent of resection. Neurosurgery.

[27] Eseonu C.I., Eguia F., ReFaey K., Garcia O., Rodriguez F.J., Chaichana K., Quinones-Hinojosa A. (2017). Comparative volumetric analysis of the extent of resection of molecularly and histologically distinct low grade gliomas and its role on survival. J. Neuro Oncol..

[28] Eseonu C.I., Rincon-Torroella J., ReFaey K., Quiñones-Hinojosa A. (2017). The cost of brain surgery: Awake vs asleep craniotomy for perirolandic region tumors. Neurosurgery.

[29] Lakomkin N., Hadjipanayis C.G. (2018). Fluorescence-guided surgery for high-grade gliomas. J. Surg. Oncol..

[30] Coburger J., Wirtz C.R. (2019). Fluorescence guided surgery by 5-ALA and intraoperative MRI in high grade glioma: A systematic review. J. Neuro Oncol..

[31] Senders J.T., Muskens I.S., Schnoor R., Karhade A.V., Cote D.J., Smith T.R., Broekman M.L. (2017). Agents for fluorescence-guided glioma surgery: A systematic review of preclinical and clinical results. Acta Neurochir..

[32] Nguyen Q.T., Tsien R.Y. (2013). Fluorescence-guided surgery with live molecular navigation—A new cutting edge. Nat. Rev. Cancer.

[33] Pirro V., Alfaro C.M., Jarmusch A.K., Hattab E.M., Cohen-Gadol A.A., Cooks R.G. (2017). Intraoperative assessment of tumor margins during glioma resection by desorption electrospray ionization-mass spectrometry. Proc. Natl. Acad. Sci. USA.

[34] Eberlin L.S., Dill A.L., Golby A.J., Ligon K.L., Wiseman J.M., Cooks R.G., Agar N.Y. (2010). Discrimination of human astrocytoma subtypes by lipid analysis using desorption electrospray ionization imaging mass spectrometry. Angew. Chem..

[35] Brown H.M., Pu F., Dey M., Miller J., Shah M.V., Shapiro S.A., Ouyang Z., Cohen-Gadol A.A., Cooks R.G. (2019). Intraoperative detection of isocitrate dehydrogenase mutations in human gliomas using a miniature mass spectrometer. Anal. Bioanal. Chem..

[36] Young R.M., Jamshidi A., Davis G., Sherman J.H. (2015). Current trends in the surgical management and treatment of adult glioblastoma. Ann. Transl. Med..

[37] Alfaro C.M., Pirro V., Keating M.F., Hattab E.M., Cooks R.G., Cohen-Gadol A.A. (2019). Intraoperative assessment of isocitrate dehydrogenase mutation status in human gliomas using desorption electrospray ionization–mass spectrometry. J. Neurosurg..

[38] Ashby L.S., Smith K.A., Stea B. (2016). Gliadel wafer implantation combined with standard radiotherapy and concurrent followed by adjuvant temozolomide for treatment of newly diagnosed high-grade glioma: A systematic literature review. World J. Surg. Oncol..

[39] Champeaux C., Weller J. (2020). Implantation of carmustine wafers (Gliadel^®^) for high-grade glioma treatment. A 9-year nationwide retrospective study. J. Neuro Oncol..

[40] Fernandes C., Costa A., Osório L., Lago R.C., Linhares P., Carvalho B., Caeiro C. (2017). Current standards of care in glioblastoma therapy. Exon Publ..

[41] Jakobsen J., Urup T., Grunnet K., Toft A., Johansen M., Poulsen S., Christensen I., Muhic A., Poulsen H. (2018). Toxicity and efficacy of lomustine and bevacizumab in recurrent glioblastoma patients. J. Neuro Oncol..

[42] Parasramka S., Talari G., Rosenfeld M., Guo J., Villano J.L. (2017). Procarbazine, lomustine and vincristine for recurrent high-grade glioma. Cochrane Database Syst. Rev..

[43] Weller M., van Den Bent M., Tonn J.C., Stupp R., Preusser M., Cohen-Jonathan-Moyal E., Henriksson R., Rhun E.L., Balana C., Chinot O. (2017). European Association for Neuro-Oncology (EANO) guideline on the diagnosis and treatment of adult astrocytic and oligodendroglial gliomas. Lancet Oncol..

[44] Weller M., Le Rhun E. (2020). How did lomustine become standard of care in recurrent glioblastoma?. Cancer Treat. Rev..

[45] Wheeler G.P., Bowdon B.J., Struck R.F. (1975). Carbamoylation of Amino Acids, Peptides, and Proteins by Nitrosoureas. Cancer Res..

[46] Kohn K.W. (1977). Interstrand cross-linking of DNA by 1,3-bis(2-chloroethyl)-1-nitrosourea and other 1-(2-haloethyl)-1-nitrosoureas. Cancer Res..

[47] Herrlinger U., Tzaridis T., Mack F., Steinbach J.P., Schlegel U., Sabel M., Hau P., Kortmann R.-D., Krex D., Grauer O. (2019). Lomustine-temozolomide combination therapy versus standard temozolomide therapy in patients with newly diagnosed glioblastoma with methylated MGMT promoter (CeTeG/NOA–09): A randomised, open-label, phase 3 trial. Lancet.

[48] Stritzelberger J., Distel L., Buslei R., Fietkau R., Putz F. (2018). Acquired temozolomide resistance in human glioblastoma cell line U251 is caused by mismatch repair deficiency and can be overcome by lomustine. Clin. Transl. Oncol..

[49] Wick W., Puduvalli V.K., Chamberlain M.C., van Den Bent M.J., Carpentier A.F., Cher L.M., Mason W., Weller M., Hong S., Musib L. (2010). Phase III study of enzastaurin compared with lomustine in the treatment of recurrent intracranial glioblastoma. J. Clin. Oncol..

[50] Yung W.K.A., Albright R.E., Olson J., Fredericks R., Fink K., Prados M.D., Brada M., Spence A., Hohl R.J., Shapiro W. (2000). A phase II study of temozolomide vs. procarbazine in patients with glioblastoma multiforme at first relapse. Br. J. Cancer.

[51] Wesolowski J.R., Rajdev P., Mukherji S.K. (2010). Temozolomide (Temodar). Ajnr Am. J. Neuroradiol..

[52] Belter A., Barciszewski J., Barciszewska A.-M. (2020). Revealing the epigenetic effect of temozolomide on glioblastoma cell lines in therapeutic conditions. PLoS ONE.

[53] Koukourakis G.V., Kouloulias V., Zacharias G., Papadimitriou C., Pantelakos P., Maravelis G., Fotineas A., Beli I., Chaldeopoulos D., Kouvaris J. (2009). Temozolomide with radiation therapy in high grade brain gliomas: Pharmaceuticals considerations and efficacy; a review article. Molecules.

[54] Schreck K.C., Grossman S.A. (2018). Role of Temozolomide in the Treatment of Cancers Involving the Central Nervous System. Oncology.

[55] Portnow J., Badie B., Chen M., Liu A., Blanchard S., Synold T.W. (2009). The Neuropharmacokinetics of Temozolomide in Patients with Resectable Brain Tumors: Potential Implications for the Current Approach to Chemoradiation. Clin. Cancer Res..

[56] Ostermann S. (2004). Plasma and Cerebrospinal Fluid Population Pharmacokinetics of Temozolomide in Malignant Glioma Patients. Clin. Cancer Res..

[57] Zhang J., FGStevens M., DBradshaw T. (2012). Temozolomide: Mechanisms of Action, Repair and Resistance. Curr. Mol. Pharmacol..

[58] Malmström A., Poulsen H.S., Grønberg B.H., Stragliotto G., Hansen S., Asklund T., Holmlund B., Łysiak M., Dowsett J., Kristensen B.W. (2017). Postoperative neoadjuvant temozolomide before radiotherapy versus standard radiotherapy in patients 60 years or younger with anaplastic astrocytoma or glioblastoma: A randomized trial. Acta Oncol..

[59] Jiapaer S., Furuta T., Tanaka S., Kitabayashi T., Nakada M. (2018). Potential strategies overcoming the temozolomide resistance for glioblastoma. Neurol. Med. Chir..

[60] Lee S.Y. (2016). Temozolomide resistance in glioblastoma multiforme. Genes Dis..

[61] Duwa R., Emami F., Lee S., Jeong J.-H., Yook S. (2019). Polymeric and lipid-based drug delivery systems for treatment of glioblastoma multiforme. J. Ind. Eng. Chem..

[62] Grimm S.A., Chamberlain M.C. (2016). Anaplastic astrocytoma. CNS Oncol..

[63] McTyre E., Lucas J.T., Helis C., Farris M., Soike M., Mott R., Laxton A.W., Tatter S.B., Lesser G.J., Strowd R.E. (2018). Outcomes for anaplastic glioma treated with radiation therapy with or without concurrent temozolomide. Am. J. Clin. Oncol..

[64] Omar A.I., Mason W.P. (2009). Temozolomide: The evidence for its therapeutic efficacy in malignant astrocytomas. Core Evid..

[65] Wei W., Chen X., Ma X., Wang D., Guo Z. (2015). The efficacy and safety of various dose-dense regimens of temozolomide for recurrent high-grade glioma: A systematic review with meta-analysis. J. Neuro Oncol..

[66] Garcia C.R., Slone S.A., Morgan R.M., Gruber L., Kumar S.S., Lightner D.D., Villano J.L. (2018). Dose-dense temozolomide for recurrent high-grade gliomas: A single-center retrospective study. Med. Oncol..

[67] Ruff M.W., Buckner J.C. (2019). The Use of PCV Chemotherapy in Oligodendrogliomas.

[68] Hafazalla K., Sahgal A., Jaja B., Perry J.R., Das S. (2018). Procarbazine, CCNU and vincristine (PCV) versus temozolomide chemotherapy for patients with low-grade glioma: A systematic review. Oncotarget.

[69] Wei W., Jia Y., Hui C. (2017). Radiotherapy plus procarbazine, lomustine, and vincristine versus radiotherapy alone for glioma: A meta-analysis of randomized controlled trials. Int. J. Clin. Exp. Med..

[70] Ruff M.W., Uhm J. (2018). Anaplastic glioma: Treatment approaches in the era of molecular diagnostics. Curr. Treat. Options Oncol..

[71] Ruff M.W., Buckner J.C., Johnson D.R., Van Den Bent M.J., Geurts M. (2019). Neuro-Oncology Clinical Debate: PCV or temozolomide in combination with radiation for newly diagnosed high-grade oligodendroglioma. Neuro Oncol. Pract..

[72] González-Aguilar A., Reyes-Moreno I., Peiro-Osuna R.P., Hernández-Hernández A., Gutiérrez-Aceves A., Santos-Zambrano J., Guerrero-Juárez V., López-Martínez M., Castro-Martínez E. (2018). Radiotherapy plus temozolomide or PCV in patients with anaplastic oligodendroglioma 1p19q codeleted. Rev. Neurol..

[73] Iwadate Y., Matsutani T., Hara A., Hirono S., Ikegami S., Kobayashi M., Ito D., Kawauchi D., Horiguchi K., Tamiya A. (2019). Eighty percent survival rate at 15 years for 1p/19q co-deleted oligodendroglioma treated with upfront chemotherapy irrespective of tumor grade. J. Neuro Oncol..

[74] McNamara M.G., Sahebjam S., Mason W.P. (2013). Anaplastic oligodendroglioma: Advances and treatment options. Curr. Treat. Options Neurol..

[75] Blakeley J., Grossman S. (2008). Anaplastic oligodendroglioma. Curr. Treat. Options Neurol..

[76] Alba A.B., Alicia T., Giovanna C., Michele R., Enrico F., Laura B., Roberta B., Marina G., Claudio G., Paolo I. (2006). Correlations Between O6-Methylguanine DNA Methyltransferase Promoter Methylation Status, 1p and 19q Deletions, and Response to Temozolomide in Anaplastic and Recurrent Oligodendroglioma: A Prospective GICNO Study. J. Clin. Oncol..

[77] Boots-Sprenger S.H., Sijben A., Rijntjes J., Tops B.B., Idema A.J., Rivera A.L., Bleeker F.E., Gijtenbeek A.M., Diefes K., Heathcock L. (2013). Significance of complete 1p/19q co-deletion, IDH1 mutation and MGMT promoter methylation in gliomas: Use with caution. Mod. Pathol..

[78] Bell E.H., Zhang P., Fisher B.J., Macdonald D.R., McElroy J.P., Lesser G.J., Fleming J., Chakraborty A.R., Liu Z., Becker A.P. (2018). Association of MGMT Promoter Methylation Status With Survival Outcomes in Patients With High-Risk Glioma Treated With Radiotherapy and Temozolomide: An Analysis From the NRG Oncology/RTOG 0424 Trial. JAMA Oncol..

[79] Gwak H.-S., Yee G.T., Park C.-K., Kim J.W., Hong Y.-K., Kang S.-G., Kim J.H., Seol H.J., Jung T.-Y., Chang J.H. (2013). Temozolomide salvage chemotherapy for recurrent anaplastic oligodendroglioma and oligo-astrocytoma. J. Korean Neurosurg. Soc..

[80] Chinot O.-L., Honore S., Dufour H., Barrie M., Figarella-Branger D., Muracciole X., Braguer D., Martin P.-M., Grisoli F. (2001). Safety and efficacy of temozolomide in patients with recurrent anaplastic oligodendrogliomas after standard radiotherapy and chemotherapy. J. Clin. Oncol..

[81] Lukas R.V., Wainwright D.A., Ladomersky E., Sachdev S., Sonabend A.M., Stupp R. (2019). Newly Diagnosed Glioblastoma: A Review on Clinical Management. Oncology.

[82] Stupp R., Mason W.P., van Den Bent M.J., Weller M., Fisher B., Taphoorn M.J.B., Belanger K., Brandes A.A., Marosi C., Bogdahn U. (2005). Radiotherapy plus Concomitant and Adjuvant Temozolomide for Glioblastoma. N. Engl. J. Med..

[83] Szopa W., Burley T.A., Kramer-Marek G., Kaspera W. (2017). Diagnostic and Therapeutic Biomarkers in Glioblastoma: Current Status and Future Perspectives. BioMed Res. Int..

[84] Ozdemir-Kaynak E., Qutub A.A., Yesil-Celiktas O. (2018). Advances in Glioblastoma Multiforme Treatment: New Models for Nanoparticle Therapy. Front. Physiol..

[85] Wick W., Roth P., Hartmann C., Hau P., Nakamura M., Stockhammer F., Sabel M.C., Wick A., Koeppen S., Ketter R. (2016). Long-term analysis of the NOA-04 randomized phase III trial of sequential radiochemotherapy of anaplastic glioma with PCV or temozolomide. Neuro Oncol..

[86] Yang P., Zhang W., Wang Y., Peng X., Chen B., Qiu X., Li G., Li S., Wu C., Yao K. (2015). IDH mutation and MGMT promoter methylation in glioblastoma: Results of a prospective registry. Oncotarget.

[87] Janaki M., Arunmohan P., Harshitha M.J. (2017). Improved Survival In A Patient Of Anaplastic Astrocytoma With Re-Irradiation: A Case Report. J. Cancer Res. Ther..

[88] Krauze A.V., Attia A., Braunstein S., Chan M., Combs S.E., Fietkau R., Fiveash J., Flickinger J., Grosu A., Howard S. (2017). Expert consensus on re-irradiation for recurrent glioma. Radiat. Oncol..

[89] Cairncross J.G., Wang M., Jenkins R.B., Shaw E.G., Giannini C., Brachman D.G., Buckner J.C., Fink K.L., Souhami L., Laperriere N.J. (2014). Benefit from procarbazine, lomustine, and vincristine in oligodendroglial tumors is associated with mutation of IDH. J. Clin. Oncol..

[90] Speirs C.K., Simpson J.R., Robinson C.G., DeWees T.A., Tran D.D., Linette G., Chicoine M.R., Dacey R.G., Rich K.M., Dowling J.L. (2015). Impact of 1p/19q codeletion and histology on outcomes of anaplastic gliomas treated with radiation therapy and temozolomide. Int. J. Radiat. Oncol. Biol. Phys..

[91] Sarmiento J.M., Venteicher A.S., Patil C.G. (2015). Early versus delayed postoperative radiotherapy for treatment of low-grade gliomas. Cochrane Database Syst. Rev..

[92] Dhawan S., Patil C.G., Chen C., Venteicher A.S. (2020). Early versus delayed postoperative radiotherapy for treatment of low-grade gliomas. Cochrane Database Syst. Rev..

[93] Im J.H., Hong J.B., Kim S.H., Choi J., Chang J.H., Cho J., Suh C.-O. (2018). Recurrence patterns after maximal surgical resection and postoperative radiotherapy in anaplastic gliomas according to the new 2016 WHO classification. Sci. Rep..

[94] Torensma R. (2018). The dilemma of cure and damage in oligodendroglioma: Ways to tip the balance away from the damage. Cancers.

[95] Venteicher A.S., Tirosh I., Hebert C., Yizhak K., Neftel C., Filbin M.G., Hovestadt V., Escalante L.E., Shaw M.L., Rodman C. (2017). Decoupling genetics, lineages, and microenvironment in IDH-mutant gliomas by single-cell RNA-seq. Science.

[96] Zhao J. (2016). Cancer stem cells and chemoresistance: The smartest survives the raid. Pharmacol. Ther..

[97] Anderson M.D., Gilbert M.R. (2014). Clinical discussion of the management of anaplastic oligodendroglioma/oligoastrocytoma (both codeleted and nondeleted). J. Natl. Compr Canc Netw..

[98] Lassman A.B. (2015). Procarbazine, lomustine and vincristine or temozolomide: Which is the better regimen?. CNS Oncol..

[99] Van Den Bent J.M. (2015). Chemotherapy for low-grade glioma: When, for whom, which regimen?. Curr. Opin. Neurol..

[100] Wick W., Hartmann C., Engel C., Stoffels M., Felsberg J., Stockhammer F., Sabel M.C., Koeppen S., Ketter R., Meyermann R. (2009). NOA-04 randomized phase III trial of sequential radiochemotherapy of anaplastic glioma with procarbazine, lomustine, and vincristine or temozolomide. J. Clin. Oncol..

[101] Wick W., Roth P., Wiestler B., Hartmann C., Hau P., Nakamura M., Stockhammer F., Sabel M., Koeppen S., Ketter R. (2015). Long-term analysis of the NOA-04 randomized phase III trial of sequential radiochemotherapy of anaplastic glioma with PCV or temozolomide. J. Clin. Oncol..

[102] Wick W., Winkler F. (2018). Regimen of procarbazine, lomustine, and vincristine versus temozolomide for gliomas. Cancer.

[103] Christians A., Adel-Horowski A., Banan R., Lehmann U., Bartels S., Behling F., Barrantes-Freer A., Stadelmann C., Rohde V., Stockhammer F. (2019). The prognostic role of IDH mutations in homogeneously treated patients with anaplastic astrocytomas and glioblastomas. Acta Neuropathol. Commun..

[104] Picca A., Berzero G., Sanson M. (2018). Current therapeutic approaches to diffuse grade II and III gliomas. Ther. Adv. Neurol. Disord..

[105] Waitkus M.S., Diplas B.H., Yan H. (2016). Isocitrate dehydrogenase mutations in gliomas. Neuro Oncol..

[106] Xu W., Yang H., Liu Y., Yang Y., Wang P., Kim S.-H., Ito S., Yang C., Wang P., Xiao M.-T. (2011). Oncometabolite 2-hydroxyglutarate is a competitive inhibitor of α-ketoglutarate-dependent dioxygenases. Cancer Cell.

[107] Liu Y., Lang F., Chou F.-J., Zaghloul K.A., Yang C. (2020). Isocitrate Dehydrogenase Mutations in Glioma: Genetics, Biochemistry, and Clinical Indications. Biomedicines.

[108] Lu C., Ward P.S., Kapoor G.S., Rohle D., Turcan S., Abdel-Wahab O., Edwards C.R., Khanin R., Figueroa M.E., Melnick A. (2012). IDH mutation impairs histone demethylation and results in a block to cell differentiation. Nature.

[109] Wang P., Wu J., Ma S., Zhang L., Yao J., Hoadley K.A., Wilkerson M.D., Perou C.M., Guan K.-L., Ye D. (2015). Oncometabolite D-2-hydroxyglutarate inhibits ALKBH DNA repair enzymes and sensitizes IDH mutant cells to alkylating agents. Cell Rep..

[110] Inoue S., Li W.Y., Tseng A., Beerman I., Elia A.J., Bendall S.C., Lemonnier F., Kron K.J., Cescon D.W., Hao Z. (2016). Mutant IDH1 downregulates ATM and alters DNA repair and sensitivity to DNA damage independent of TET2. Cancer Cell.

[111] Pellerino A., Bertero L., Rudà R., Soffietti R. (2020). Choosing appropriate chemotherapy for diffusely infiltrating WHO grade II gliomas in adults. Expert Opin. Pharmacother..

[112] Valiyaveettil D., Malik M., Joseph D., Ahmed S.F., Kothwal S.A. (2018). Prognostic factors and outcomes in anaplastic gliomas: An institutional experience. S. Asian J. Cancer.

[113] Jovanović N., Mitrović T., Cvetković V.J., Tošić S., Vitorović J., Stamenković S., Nikolov V., Kostić A., Vidović N., Krstić M. (2019). The Impact of Promoter Methylation and Temozolomide Treatment in Serbian Patients with Primary Glioblastoma. Medicina.

[114] Yi G.Z., Huang G., Guo M., Zhang X., Wang H., Deng S., Li Y., Xiang W., Chen Z., Pan J. (2019). Acquired temozolomide resistance in MGMT-deficient glioblastoma cells is associated with regulation of DNA repair by DHC2. Brain.

[115] Bienkowski M., Berghoff A.S., Marosi C., Wöhrer A., Heinzl H., Hainfellner J.A., Preusser M. (2015). Clinical Neuropathology practice guide 5–2015: MGMT methylation pyrosequencing in glioblastoma: Unresolved issues and open questions. Clin. Neuropathol..

[116] Malley D.S., Hamoudi R.A., Kocialkowski S., Pearson D.M., Collins V.P., Ichimura K. (2011). A distinct region of the MGMT CpG island critical for transcriptional regulation is preferentially methylated in glioblastoma cells and xenografts. Acta Neuropathol..

[117] Mansouri A., Hachem L.D., Mansouri S., Nassiri F., Laperriere N.J., Xia D., Lindeman N.I., Wen P.Y., Chakravarti A., Mehta M.P. (2019). MGMT promoter methylation status testing to guide therapy for glioblastoma: Refining the approach based on emerging evidence and current challenges. Neuro Oncol..

[118] Hegi M.E., Liu L., Herman J.G., Stupp R., Wick W., Weller M., Mehta M.P., Gilbert M.R. (2008). Correlation of O6-methylguanine methyltransferase (MGMT) promoter methylation with clinical outcomes in glioblastoma and clinical strategies to modulate MGMT activity. J. Clin. Oncol..

[119] Hombach-Klonisch S., Mehrpour M., Shojaei S., Harlos C., Pitz M., Hamai A., Siemianowicz K., Likus W., Wiechec E., Toyota B.D. (2018). Glioblastoma and chemoresistance to alkylating agents: Involvement of apoptosis, autophagy, and unfolded protein response. Pharmacol. Ther..

[120] Li Q., Guo J., Wang W., Wang D. (2017). Relationship between MGMT gene expression and treatment effectiveness and prognosis in glioma. Oncol. Lett..

[121] Rabé M., Dumont S., Álvarez-Arenas A., Janati H., Belmonte-Beitia J., Calvo G.F., Thibault-Carpentier C., Séry Q., Chauvin C., Joalland N. (2020). Identification of a transient state during the acquisition of temozolomide resistance in glioblastoma. Cell Death Dis..

[122] Woo P., Li Y., Chan A., Ng S., Loong H., Chan D., Wong G., Poon W.-S. (2019). A multifaceted review of temozolomide resistance mechanisms in glioblastoma beyond O-6-methylguanine-DNA methyltransferase. Glioma.

[123] Chen X., Zhang M., Gan H., Wang H., Jeong-Heon L., Fang D., Kitange G., He L., Hu Z., Parney I. (2018). A novel enhancer regulates MGMT expression and promotes temozolomide resistance in glioblastoma. Nat. Commun..

[124] Low S.Y.Y., Ho Y.K., Too H.-P., Yap C.T., Ng W.H. (2014). MicroRNA as potential modulators in chemoresistant high-grade gliomas. J. Clin. Neurosci..

[125] Kaina B., Christmann M., Naumann S., Roos W.P. (2007). MGMT: Key node in the battle against genotoxicity, carcinogenicity and apoptosis induced by alkylating agents. DNA Repair.

[126] Aasland D., Götzinger L., Hauck L., Berte N., Meyer J., Effenberger M., Schneider S., Reuber E.E., Roos W.P., Tomicic M.T. (2019). Temozolomide Induces Senescence and Repression of DNA Repair Pathways in Glioblastoma Cells via Activation of ATR-CHK1, p21, and NF-κB. Cancer Res..

[127] McFaline-Figueroa J.L., Braun C.J., Stanciu M., Nagel Z.D., Mazzucato P., Sangaraju D., Cerniauskas E., Barford K., Vargas A., Chen Y. (2015). Minor Changes in Expression of the Mismatch Repair Protein MSH2 Exert a Major Impact on Glioblastoma Response to Temozolomide. Cancer Res..

[128] Erasimus H., Gobin M., Niclou S., Van Dyck E. (2016). DNA repair mechanisms and their clinical impact in glioblastoma. Mutat. Res. Rev. Mutat. Res..

[129] Atkins R.J., Ng W., Stylli S.S., Hovens C.M., Kaye A.H. (2015). Repair mechanisms help glioblastoma resist treatment. J. Clin. Neurosci..

[130] Angeli J., Krysko D.V., Conrad M. (2019). Ferroptosis at the crossroads of cancer-acquired drug resistance and immune evasion. Nat. Rev. Cancer.

[131] Gao X., Guo N., Xu H., Pan T., Lei H., Yan A., Mi Y., Xu L. (2020). Ibuprofen induces ferroptosis of glioblastoma cells via downregulation of nuclear factor erythroid 2-related factor 2 signaling pathway. Anti Cancer Drugs.

[132] Hirose Y., Berger M.S., Pieper R.O. (2001). p53 effects both the duration of G 2 /M arrest and the fate of temozolomide-treated human glioblastoma cells. Cancer Res..

[133] Chien C.-H., Hsueh W.-T., Chuang J.-Y., Chang K.-Y. (2019). Role of autophagy in therapeutic resistance of glioblastoma. J. Cancer Metastas. Treat..

[134] Buccarelli M., Marconi M., Pacioni S., De Pascalis I., D’Alessandris Q.G., Martini M., Ascione B., Malorni W., Larocca L.M., Pallini R. (2018). Inhibition of autophagy increases susceptibility of glioblastoma stem cells to temozolomide by igniting ferroptosis. Cell Death Dis..

[135] Yang K., Niu L., Bai Y., Le W. (2019). Glioblastoma: Targeting the autophagy in tumorigenesis. Brain Res. Bull..

[136] Das C.K., Mandal M., Kögel D. (2018). Pro-survival autophagy and cancer cell resistance to therapy. Cancer Metastas. Rev..

[137] Ambrosio S., Majello B. (2020). Autophagy Roles in Genome Maintenance. Cancers.

[138] Huang W.J., Chen W.W., Zhang X. (2016). Glioblastoma multiforme: Effect of hypoxia and hypoxia inducible factors on therapeutic approaches. Oncol. Lett..

[139] Graham K., Unger E. (2018). Overcoming tumor hypoxia as a barrier to radiotherapy, chemotherapy and immunotherapy in cancer treatment. Int. J. Nanomed..

[140] Colwell N., Larion M., Giles A.J., Seldomridge A.N., Sizdahkhani S., Gilbert M.R., Park D.M. (2017). Hypoxia in the glioblastoma microenvironment: Shaping the phenotype of cancer stem-like cells. Neuro Oncol..

[141] Monteiro A.R., Hill R., Pilkington G.J., Madureira P.A. (2017). The role of hypoxia in glioblastoma invasion. Cells.

[142] Lo Dico A., Martelli C., Diceglie C., Lucignani G., Ottobrini L. (2018). Hypoxia-inducible factor-1α activity as a switch for glioblastoma responsiveness to temozolomide. Front. Oncol..

[143] Li P., Zhang D., Shen L., Dong K., Wu M., Ou Z., Shi D. (2016). Redox homeostasis protects mitochondria through accelerating ROS conversion to enhance hypoxia resistance in cancer cells. Sci. Rep..

[144] Chen R., Lai U.H., Zhu L., Singh A., Ahmed M., Forsyth N.R. (2018). Reactive oxygen species formation in the brain at different oxygen levels: The role of hypoxia inducible factors. Front. Cell Dev. Biol..

[145] Wang H., Jiang H., Van De Gucht M., De Ridder M. (2019). Hypoxic radioresistance: Can ROS be the key to overcome it?. Cancers.

[146] Stępień K., Ostrowski R.P., Matyja E. (2016). Hyperbaric oxygen as an adjunctive therapy in treatment of malignancies, including brain tumours. Med. Oncol..

[147] Yahara K., Ohguri T., Udono H., Yamamoto J., Tomura K., Onoda T., Imada H., Nishizawa S., Korogi Y. (2017). Radiotherapy using IMRT boosts after hyperbaric oxygen therapy with chemotherapy for glioblastoma. J. Radiat. Res..

[148] Buehler H., Strohm G.L., Nguemgo-Kouam P., Lamm H., Fakhrian K., Adamietz I.A. (2015). The therapeutic effect of photon irradiation on viable glioblastoma cells is reinforced by hyperbaric oxygen. Anticancer Res..

[149] Clarke R.H., Moosa S., Anzivino M., Wang Y., Floyd D.H., Purow B.W., Lee K.S. (2014). Sustained radiosensitization of hypoxic glioma cells after oxygen pretreatment in an animal model of glioblastoma and in vitro models of tumor hypoxia. PLoS ONE.

[150] Lu Z., Ma J., Liu B., Dai C., Xie T., Ma X., Li M., Dong J., Lan Q., Huang Q. (2016). Hyperbaric oxygen therapy sensitizes nimustine treatment for glioma in mice. Cancer Med..

[151] Xie Y., Zeng X., Wu X., Hu J., Zhu Y., Yang X. (2018). Hyperbaric oxygen as an adjuvant to temozolomide nanoparticle inhibits glioma growth by inducing G2/M phase arrest. Nanomedicine.

[152] Kumar G., Dutta P., Parihar V.K., Chamallamudi M.R., Kumar N. (2020). Radiotherapy and Its Impact on the Nervous System of Cancer Survivors. CNS Neurol. Disord. Drug Targets.

[153] Toussaint L., Indelicato D.J., Stokkevåg C.H., Lassen-Ramshad Y., Pedro C., Mikkelsen R., Di Pinto M., Li Z., Flampouri S., Vestergaard A. (2019). Radiation doses to brain substructures associated with cognition in radiotherapy of pediatric brain tumors. Acta Oncol..

[154] Makale M.T., McDonald C.R., Hattangadi-Gluth J.A., Kesari S. (2017). Mechanisms of radiotherapy-associated cognitive disability in patients with brain tumours. Nat. Rev. Neurol..

[155] Ghia A.J. (2018). Fractionated radiotherapy of intracranial gliomas. Intracranial Gliomas Part II-Adjuvant Therapy.

[156] Song A., Andrews D.W., Werner-Wasik M., Kim L., Glass J., Bar-Ad V., Evans J.J., Farrell C.J., Judy K.D., Daskalakis C. (2019). Phase I trial of alisertib with concurrent fractionated stereotactic re-irradiation for recurrent high grade gliomas. Radiother. Oncol..

[157] Nachbichler S.B., Kreth F.-W. (2018). Brachytherapy of intracranial gliomas. Intracranial Gliomas Part II-Adjuvant Therapy.

[158] Bartek J., Alattar A.A., Dhawan S., Ma J., Koga T., Nakaji P., Dusenbery K.E., Chen C.C. (2019). Receipt of brachytherapy is an independent predictor of survival in glioblastoma in the Surveillance, Epidemiology, and End Results database. J. Neuro Oncol..

[159] Barbarite E., Sick J.T., Berchmans E., Bregy A., Shah A.H., Elsayyad N., Komotar R.J. (2017). The role of brachytherapy in the treatment of glioblastoma multiforme. Neurosurg. Rev..

[160] Cabrera A., Kirkpatrick J., Fiveash J., Shih H., Koay E., Lutz S., Reardon D., Petit J., Chao S., Brown P. (2016). Radiation therapy for glioblastoma: An astro evidence-based clinical practice guideline. Pract. Radiat Oncol..

[161] Barney C., Shukla G., Bhamidipati D., Palmer J.D. (2017). Re-irradiation for recurrent glioblastoma multiforme. Chin. Clin. Oncol..

[162] Darakchiev B.J., Albright R.E., Breneman J.C., Warnick R.E. (2008). Safety and efficacy of permanent iodine-125 seed implants and carmustine wafers in patients with recurrent glioblastoma multiforme. J. Neurosurg..

[163] Caffery B., Lee J.S., Alexander-Bryant A.A. (2019). Vectors for glioblastoma gene therapy: Viral & non-viral delivery strategies. Nanomaterials.

[164] Hossain J.A., Marchini A., Fehse B., Bjerkvig R., Miletic H. (2020). Suicide gene therapy for the treatment of high-grade glioma: Past lessons, present trends, and future prospects. Neuro Oncol. Adv..

[165] Portnow J., Synold T.W., Badie B., Tirughana R., Lacey S.F., D’Apuzzo M., Metz M.Z., Najbauer J., Bedell V., Vo T. (2017). Neural stem cell–based anticancer gene therapy: A first-in-human study in recurrent high-grade glioma patients. Clin. Cancer Res..

[166] Chen S.-H., Sun J.-M., Chen B.-M., Lin S.-C., Chang H.-F., Collins S., Chang D., Wu S.-F., Lu Y.-C., Wang W. (2020). Efficient prodrug activator gene therapy by retroviral replicating vectors prolongs survival in an immune-competent intracerebral glioma model. Int. J. Mol. Sci..

[167] Manikandan C., Kaushik A., Sen D. (2019). Viral vector: Potential therapeutic for glioblastoma multiforme. Cancer Gene Ther..

[168] Artene S.-A., Turcu-Stiolica A., Ciurea M.E., Folcuti C., Tataranu L.G., Alexandru O., Purcaru O.S., Tache D.E., Boldeanu M.V., Silosi C. (2018). Comparative effect of immunotherapy and standard therapy in patients with high grade glioma: A meta-analysis of published clinical trials. Sci. Rep..

[169] Nduom E.K., Weller M., Heimberger A.B. (2015). Immunosuppressive mechanisms in glioblastoma. Neuro Oncol..

[170] Grabowski M.M., Sankey E.W., Ryan K.J., Chongsathidkiet P., Lorrey S.J., Wilkinson D.S., Fecci P.E. (2020). Immune suppression in gliomas. J. Neuro Oncol..

[171] Hossain J.A., Latif M.A., Ystaas L.A., Ninzima S., Riecken K., Muller A., Azuaje F., Joseph J.V., Talasila K.M., Ghimire J. (2019). Long-term treatment with valganciclovir improves lentiviral suicide gene therapy of glioblastoma. Neuro Oncol..

[172] Stedt H., Samaranayake H., Kurkipuro J., Wirth G., Christiansen L., Vuorio T., Määttä A., Piškur J., Ylä-Herttuala S. (2015). Tomato thymidine kinase-based suicide gene therapy for malignant glioma—An alternative for Herpes Simplex virus-1 thymidine kinase. Cancer Gene Ther..

[173] Dührsen L., Hartfuß S., Hirsch D., Geiger S., Maire C.L., Sedlacik J., Guenther C., Westphal M., Lamszus K., Hermann F.G. (2019). Preclinical analysis of human mesenchymal stem cells: Tumor tropism and therapeutic efficiency of local HSV-TK suicide gene therapy in glioblastoma. Oncotarget.

[174] Huang T.T., Parab S., Burnett R., Diago O., Ostertag D., Hofman F.M., Espinoza F.L., Martin B., Ibanez C.E., Kasahara N. (2015). Intravenous administration of retroviral replicating vector, Toca 511, demonstrates therapeutic efficacy in orthotopic immune-competent mouse glioma model. Hum. Gene Ther..

[175] Mitchell L.A., Lopez Espinoza F., Mendoza D., Kato Y., Inagaki A., Hiraoka K., Kasahara N., Gruber H.E., Jolly D.J., Robbins J.M. (2017). Toca 511 gene transfer and treatment with the prodrug, 5-fluorocytosine, promotes durable antitumor immunity in a mouse glioma model. Neuro Oncol..

[176] Philbrick B.D., Adamson D.C. (2019). Early clinical trials of Toca 511 and Toca FC show a promising novel treatment for recurrent malignant glioma. Expert Opin. Investig. Drugs.

[177] Accomando W.P., Rao A.R., Hogan D.J., Newman A.M., Nakao A., Alizadeh A.A., Diehn M., Diago O.R., Gammon D.K., Haghighi A. (2020). Molecular and immunological signatures are related to clinical benefit from treatment with Vocimagene amiretrorepvec (Toca 511) and 5-fluorocytosine (Toca FC) in patients with glioma. Clin. Cancer Res..

[178] Yagiz K., Huang T.T., Lopez Espinoza F., Mendoza D., Ibanez C.E., Gruber H.E., Jolly D.J., Robbins J.M. (2016). Toca 511 plus 5-fluorocytosine in combination with lomustine shows chemotoxic and immunotherapeutic activity with no additive toxicity in rodent glioblastoma models. Neuro Oncol..

[179] Lee M., Kim Y.-S., Lee K., Kang M., Shin H., Oh J.-W., Koo H., Kim D., Kim Y., Kong D.-S. (2019). Novel Semi-Replicative Retroviral Vector Mediated Double Suicide Gene Transfer Enhances Antitumor Effects in Patient-Derived Glioblastoma Models. Cancers.

[180] Ji N., Weng D., Liu C., Gu Z., Chen S., Guo Y., Fan Z., Wang X., Chen J., Zhao Y. (2016). Adenovirus-mediated delivery of herpes simplex virus thymidine kinase administration improves outcome of recurrent high-grade glioma. Oncotarget.

[181] Shimazu Y., Kurozumi K., Ichikawa T., Fujii K., Onishi M., Ishida J., Oka T., Watanabe M., Nasu Y., Kumon H. (2015). Integrin antagonist augments the therapeutic effect of adenovirus-mediated REIC/Dkk-3 gene therapy for malignant glioma. Gene Ther..

[182] Kiyokawa J., Wakimoto H. (2019). Preclinical and clinical development of oncolytic adenovirus for the treatment of malignant glioma. Oncolytic Virother..

[183] Nan Y., Guo L., Song Y., Wang L., Yu K., Huang Q., Zhong Y. (2017). Combinatorial therapy with adenoviral-mediated PTEN and a PI3K inhibitor suppresses malignant glioma cell growth in vitro and in vivo by regulating the PI3K/AKT signaling pathway. J. Cancer Res. Clin. Oncol..

[184] Rainov N.G. (2000). A phase III clinical evaluation of herpes simplex virus type 1 thymidine kinase and ganciclovir gene therapy as an adjuvant to surgical resection and radiation in adults with previously untreated glioblastoma multiforme. Hum. Gene Ther..

[185] Stragliotto G., Rahbar A., Solberg N.W., Lilja A., Taher C., Orrego A., Bjurman B., Tammik C., Skarman P., Peredo I. (2013). Effects of valganciclovir as an add-on therapy in patients with cytomegalovirus-positive glioblastoma: A randomized, double-blind, hypothesis-generating study. Int. J. Cancer.

[186] Westphal M., Ylä-Herttuala S., Martin J., Warnke P., Menei P., Eckland D., Kinley J., Kay R., Ram Z., Group A.S. (2013). Adenovirus-mediated gene therapy with sitimagene ceradenovec followed by intravenous ganciclovir for patients with operable high-grade glioma (ASPECT): A randomised, open-label, phase 3 trial. Lancet Oncol..

[187] Wheeler L.A., Manzanera A.G., Bell S.D., Cavaliere R., McGregor J.M., Grecula J.C., Newton H.B., Lo S.S., Badie B., Portnow J. (2016). Phase II multicenter study of gene-mediated cytotoxic immunotherapy as adjuvant to surgical resection for newly diagnosed malignant glioma. Neuro Oncol..

[188] Huang B., Zhang H., Gu L., Ye B., Jian Z., Stary C., Xiong X.X. (2017). Advances in Immunotherapy for Glioblastoma Multiforme. J. Immunol. Res..

[189] Liau L.M., Ashkan K., Tran D.D., Campian J.L., Trusheim J.E., Cobbs C.S., Heth J.A., Salacz M., Taylor S., D’Andre S.D. (2018). First results on survival from a large Phase 3 clinical trial of an autologous dendritic cell vaccine in newly diagnosed glioblastoma. J. Transl. Med..

[190] Tonigold M., Simon J., Estupiñán D., Kokkinopoulou M., Reinholz J., Kintzel U., Kaltbeitzel A., Renz P., Domogalla M.P., Steinbrink K. (2018). Pre-adsorption of antibodies enables targeting of nanocarriers despite a biomolecular corona. Nat. Nanotechnol..

[191] Loureiro J.A., Ramalho M.J., Carmo Pereira M.D. (2020). Immuno-nanocarriers for brain delivery: Limitations from in vitro to preclinical and clinical studies. Nanomedicine.

[192] Ampie L., Choy W., Lamano J.B., Fakurnejad S., Bloch O., Parsa A.T. (2015). Heat shock protein vaccines against glioblastoma: From bench to bedside. J. Neuro Oncol..

[193] Weller M., Roth P., Preusser M., Wick W., Reardon D.A., Platten M., Sampson J.H. (2017). Vaccine-based immunotherapeutic approaches to gliomas and beyond. Nat. Rev. Neurol..

[194] Rahat M.A. (2019). Targeting angiogenesis with peptide vaccines. Front. Immunol..

[195] Wen P.Y., Reardon D.A., Armstrong T.S., Phuphanich S., Aiken R.D., Landolfi J.C., Curry W.T., Zhu J.-J., Glantz M., Peereboom D.M. (2019). A randomized double-blind placebo-controlled phase II trial of dendritic cell vaccine ICT-107 in newly diagnosed patients with glioblastoma. Clin. Cancer Res..

[196] Chakraborty S., Schneider J., Boockvar J.A. (2016). Transdifferentiation induced neural stem cells for the treatment of malignant gliomas. Neurosurgery.

[197] Weller M., Butowski N., Tran D.D., Recht L.D., Lim M., Hirte H., Ashby L., Mechtler L., Goldlust S.A., Iwamoto F. (2017). Rindopepimut with temozolomide for patients with newly diagnosed, EGFRvIII-expressing glioblastoma (ACT IV): A randomised, double-blind, international phase 3 trial. Lancet Oncol..

[198] Bagley S.J., Desai A.S., Linette G.P., June C.H., O’Rourke D.M. (2018). CAR T-cell therapy for glioblastoma: Recent clinical advances and future challenges. Neuro Oncol..

[199] Li L., Zhu X., Qian Y., Yuan X., Ding Y., Hu D., He X., Wu Y. (2020). Chimeric Antigen Receptor T-Cell Therapy in Glioblastoma: Current and Future. Front. Immunol..

[200] Migliorini D., Dietrich P.-Y., Stupp R., Linette G.P., Posey A.D., June C.H. (2018). CAR T-cell therapies in glioblastoma: A first look. Clin. Cancer Res..

[201] Prinzing B.L., Gottschalk S.M., Krenciute G. (2018). CAR T-cell therapy for glioblastoma: Ready for the next round of clinical testing?. Expert Rev. Anticancer Ther..

[202] Burger M.C., Zhang C., Harter P.N., Romanski A., Strassheimer F., Senft C., Tonn T., Steinbach J.P., Wels W.S. (2019). CAR-engineered NK cells for the treatment of glioblastoma: Turning innate effectors into precision tools for cancer immunotherapy. Front. Immunol..

[203] Jin L., Ge H., Long Y., Yang C., Chang Y., Mu L., Sayour E.J., De Leon G., Wang Q.J., Yang J.C. (2018). CD70, a novel target of CAR T-cell therapy for gliomas. Neuro Oncol..

[204] Tang X., Zhao S., Zhang Y., Wang Y., Zhang Z., Yang M., Zhu Y., Zhang G., Guo G., Tong A. (2019). B7-H3 as a novel CAR-T therapeutic target for glioblastoma. Mol. Ther. Oncolytics.

[205] Li Y., Wu H., Chen G., Wei X., Wang C., Zhou S., Huang A., Zhang Z., Zhan C., Wu Y. (2020). Arming Anti-EGFRvIII CAR-T With TGFβ Trap Improves Antitumor Efficacy in Glioma Mouse Models. Front. Oncol..

[206] Bielamowicz K., Fousek K., Byrd T.T., Samaha H., Mukherjee M., Aware N., Wu M.-F., Orange J.S., Sumazin P., Man T.-K. (2018). Trivalent CAR T cells overcome interpatient antigenic variability in glioblastoma. Neuro Oncol..

[207] Weiss T., Weller M., Guckenberger M., Sentman C.L., Roth P. (2018). NKG2D-based CAR T cells and radiotherapy exert synergistic efficacy in glioblastoma. Cancer Res..

[208] Suryadevara C.M., Desai R., Abel M.L., Riccione K.A., Batich K.A., Shen S.H., Chongsathidkiet P., Gedeon P.C., Elsamadicy A.A., Snyder D.J. (2018). Temozolomide lymphodepletion enhances CAR abundance and correlates with antitumor efficacy against established glioblastoma. Oncoimmunology.

[209] Diaz R.J., Ali S., Qadir M.G., Macarena I., Ivan M.E., Komotar R.J. (2017). The role of bevacizumab in the treatment of glioblastoma. J. Neuro Oncol..

[210] Sousa F., Dhaliwal H.K., Gattacceca F., Sarmento B., Amiji M.M. (2019). Enhanced anti-angiogenic effects of bevacizumab in glioblastoma treatment upon intranasal administration in polymeric nanoparticles. J. Control. Release.

[211] Kickingereder P., Brugnara G., Hansen M.B., Nowosielski M., Pflüger I., Schell M., Isensee F., Foltyn M., Neuberger U., Kessler T. (2020). Noninvasive Characterization of Tumor Angiogenesis and Oxygenation in Bevacizumab-treated Recurrent Glioblastoma by Using Dynamic Susceptibility MRI: Secondary Analysis of the European Organization for Research and Treatment of Cancer 26101 Trial. Radiology.

[212] Clarke J., Neil E., Terziev R., Gutin P., Barani I., Kaley T., Lassman A.B., Chan T.A., Yamada J., DeAngelis L. (2017). Multicenter, phase 1, dose escalation study of hypofractionated stereotactic radiation therapy with bevacizumab for recurrent glioblastoma and anaplastic astrocytoma. Int. J. Radiat. Oncol. Biol. Phys..

[213] Kreisl T.N., Zhang W., Odia Y., Shih J.H., Butman J.A., Hammoud D., Iwamoto F.M., Sul J., Fine H.A. (2011). A phase II trial of single-agent bevacizumab in patients with recurrent anaplastic glioma. Neuro Oncol..

[214] Lee E.Q., Reardon D.A., Schiff D., Drappatz J., Muzikansky A., Grimm S.A., Norden A.D., Nayak L., Beroukhim R., Rinne M.L. (2015). Phase II study of panobinostat in combination with bevacizumab for recurrent glioblastoma and anaplastic glioma. Neuro Oncol..

[215] Chamberlain M.C., Johnston S. (2009). Salvage chemotherapy with bevacizumab for recurrent alkylator-refractory anaplastic astrocytoma. J. Neuro Oncol..

[216] Van Den Bent M., Gan H.K., Lassman A.B., Kumthekar P., Merrell R., Butowski N., Lwin Z., Mikkelsen T., Nabors L.B., Papadopoulos K.P. (2017). Efficacy of depatuxizumab mafodotin (ABT-414) monotherapy in patients with EGFR-amplified, recurrent glioblastoma: Results from a multi-center, international study. Cancer Chemother. Pharmacol..

[217] Narita Y., Muragaki Y., Maruyama T., Kagawa N., Asai K., Kuroda J., Kurozumi K., Nagane M., Matsuda M., Ueki K. (2019). Phase I/II study of depatuxizumab mafodotin (ABT-414) monotherapy or combination with temozolomide in Japanese patients with/without EGFR-amplified recurrent glioblastoma. J. Clin. Oncol..

[218] Von Achenbach C., Silginer M., Blot V., Weiss W.A., Weller M. (2020). Depatuxizumab mafodotin (ABT-414)-induced glioblastoma cell death requires EGFR overexpression, but not EGFRY1068 phosphorylation. Mol. Cancer Ther..

[219] Gatson N.T.N., Weathers S.-P.S., de Groot J.F. (2016). ReACT Phase II trial: A critical evaluation of the use of rindopepimut plus bevacizumab to treat EGFRvIII-positive recurrent glioblastoma. CNS Oncol..

[220] Reardon D.A., Desjardins A., Vredenburgh J.J., O’Rourke D.M., Tran D.D., Fink K.L., Nabors L.B., Li G., Bota D.A., Lukas R.V. (2020). Rindopepimut with bevacizumab for patients with relapsed EGFRvIII-expressing glioblastoma (ReACT): Results of a double-blind randomized phase II trial. Clin. Cancer Res..

[221] Paff M., Alexandru-Abrams D., Hsu F.P., Bota D.A. (2014). The evolution of the EGFRvIII (rindopepimut) immunotherapy for glioblastoma multiforme patients. Hum. Vaccines Immunother..

[222] Elsamadicy A.A., Chongsathidkiet P., Desai R., Woroniecka K., Farber S.H., Fecci P.E., Sampson J.H. (2017). Prospect of rindopepimut in the treatment of glioblastoma. Expert Opin. Biol. Ther..

[223] Bloch O., Crane C.A., Fuks Y., Kaur R., Aghi M.K., Berger M.S., Butowski N.A., Chang S.M., Clarke J.L., McDermott M.W. (2014). Heat-shock protein peptide complex–96 vaccination for recurrent glioblastoma: A phase II, single-arm trial. Neuro Oncol..

[224] Bloch O., Shi Q., Anderson S.K., Knopp M., Raizer J., Clarke J., Waziri A., Colman H., Bruce J., Olson J.J. (2017). ATIM-14. Alliance A071101: A phase II randomized trial comparing the efficacy of heat shock protein peptide complex-96 (HSPPC-96) vaccine given with bevacizumab versus bevacizumab alone in the treatment of surgically resectable recurrent glioblastoma. Neuro Oncol..

[225] Ji N., Zhang Y., Liu Y., Xie J., Wang Y., Hao S., Gao Z. (2018). Heat shock protein peptide complex-96 vaccination for newly diagnosed glioblastoma: A phase I, single-arm trial. JCI Insight.

[226] Srivastava S., Jackson C., Kim T., Choi J., Lim M. (2019). A characterization of dendritic cells and their role in immunotherapy in glioblastoma: From preclinical studies to clinical trials. Cancers.

[227] Malo C.S., Khadka R.H., Ayasoufi K., Jin F., AbouChehade J.E., Hansen M.J., Iezzi R., Pavelko K.D., Johnson A.J. (2018). Immunomodulation mediated by anti-angiogenic therapy improves CD8 T cell immunity against experimental glioma. Front. Oncol..

[228] Mosaheb M.M., Dobrikova E.Y., Brown M.C., Yang Y., Cable J., Okada H., Nair S.K., Bigner D.D., Ashley D.M., Gromeier M. (2020). Genetically stable poliovirus vectors activate dendritic cells and prime antitumor CD8 T cell immunity. Nat. Commun..

[229] Prins R.M., Soto H., Konkankit V., Odesa S.K., Eskin A., Yong W.H., Nelson S.F., Liau L.M. (2011). Gene expression profile correlates with T-cell infiltration and relative survival in glioblastoma patients vaccinated with dendritic cell immunotherapy. Clin. Cancer Res..

[230] Antonios J., Everson R., Soto H., Khattab S., Bethel J., Sun M., Mochizuki A., Lee A., Odesa S., Billingslea-Yoon E. (2018). Atim-39. Improved survival noted in glioblastoma patients treated with adjuvant tlr-3 agonist in setting of autologous lysate-pulsed dc vaccination. Neuro Oncol..

[231] Cho D.-Y., Yang W.-K., Lee H.-C., Hsu D.-M., Lin H.-L., Lin S.-Z., Chen C.-C., Harn H.-J., Liu C.-L., Lee W.-Y. (2012). Adjuvant immunotherapy with whole-cell lysate dendritic cells vaccine for glioblastoma multiforme: A phase II clinical trial. World Neurosurg..

[232] Wheeler C.J., Das A., Liu G., John S.Y., Black K.L. (2004). Clinical responsiveness of glioblastoma multiforme to chemotherapy after vaccination. Clin. Cancer Res..

[233] Yu J.S., Liu G., Ying H., Yong W.H., Black K.L., Wheeler C.J. (2004). Vaccination with tumor lysate-pulsed dendritic cells elicits antigen-specific, cytotoxic T-cells in patients with malignant glioma. Cancer Res..

[234] Batich K.A., Reap E.A., Archer G.E., Sanchez-Perez L., Nair S.K., Schmittling R.J., Norberg P., Xie W., Herndon J.E., Healy P. (2017). Long-term Survival in Glioblastoma with Cytomegalovirus pp65-Targeted Vaccination. Clin. Cancer Res..

[235] Chang C.-N., Huang Y.-C., Yang D.-M., Kikuta K., Wei K.-J., Kubota T., Yang W.-K. (2011). A phase I/II clinical trial investigating the adverse and therapeutic effects of a postoperative autologous dendritic cell tumor vaccine in patients with malignant glioma. J. Clin. Neurosci..

[236] Yamanaka R., Homma J., Yajima N., Tsuchiya N., Sano M., Kobayashi T., Yoshida S., Abe T., Narita M., Takahashi M. (2005). Clinical evaluation of dendritic cell vaccination for patients with recurrent glioma: Results of a clinical phase I/II trial. Clin. Cancer Res..

[237] Jie X., Hua L., Jiang W., Feng F., Feng G., Hua Z. (2012). Clinical application of a dendritic cell vaccine raised against heat-shocked glioblastoma. Cell Biochem. Biophys..

[238] Vik-Mo E.O., Nyakas M., Mikkelsen B.V., Moe M.C., Due-Tønnesen P., Suso E.M.I., Sæbøe-Larssen S., Sandberg C., Brinchmann J.E., Helseth E. (2013). Therapeutic vaccination against autologous cancer stem cells with mRNA-transfected dendritic cells in patients with glioblastoma. Cancer Immunol. Immunother..

[239] Branter J., Basu S., Smith S. (2018). Tumour treating fields in a combinational therapeutic approach. Oncotarget.

[240] Kirson E.D., Dbalý V., Tovaryš F., Vymazal J., Soustiel J.F., Itzhaki A., Mordechovich D., Steinberg-Shapira S., Gurvich Z., Schneiderman R. (2007). Alternating electric fields arrest cell proliferation in animal tumor models and human brain tumors. Proc. Natl. Acad. Sci. USA.

[241] Luo C., Xu S., Dai G., Xiao Z., Chen L., Liu Z. (2020). Tumor treating fields for high-grade gliomas. BioMed Pharm..

[242] Stupp R., Taillibert S., Kanner A., Read W., Steinberg D.M., Lhermitte B., Toms S., Idbaih A., Ahluwalia M.S., Fink K. (2017). Effect of tumor-treating fields plus maintenance temozolomide vs maintenance temozolomide alone on survival in patients with glioblastoma: A randomized clinical trial. JAMA.

[243] Guzauskas G.F., Pollom E.L., Stieber V.W., Wang B.C., Garrison L.P. (2019). Tumor treating fields and maintenance temozolomide for newly-diagnosed glioblastoma: A cost-effectiveness study. J. Med. Econ..

[244] Lu G., Rao M., Zhu P., Liang B., El-Nazer R.T., Fonkem E., Bhattacharjee M.B., Zhu J.-J. (2019). Triple-drug therapy with bevacizumab, irinotecan, and temozolomide plus tumor treating fields for recurrent glioblastoma: A retrospective study. Front. Neurol..

[245] Robins H.I., Nguyen H.N., Field A., Howard S., Salamat S., Deming D.A. (2018). Molecular evolution of a glioblastoma controlled with tumor treating fields and concomitant temozolomide. Front. Oncol..

[246] Liao C.-H., Lai I.C., Kuo H.-C., Chuang S.-E., Lee H.-L., Whang-Peng J., Yao C.-J., Lai G.-M. (2019). Epigenetic Modification and Differentiation Induction of Malignant Glioma Cells by Oligo-Fucoidan. Mar. Drugs.

[247] Guzauskas G.F., Salzberg M., Wang B.C. (2018). Estimated lifetime survival benefit of tumor treating fields and temozolomide for newly diagnosed glioblastoma patients. CNS Oncol..

[248] Fabian D., Guillermo Prieto Eibl M.D., Alnahhas I., Sebastian N., Giglio P., Puduvalli V., Gonzalez J., Palmer J.D. (2019). Treatment of Glioblastoma (GBM) with the Addition of Tumor-Treating Fields (TTF): A Review. Cancers.

[249] Bernard-Arnoux F., Lamure M., Ducray F., Aulagner G., Honnorat J., Armoiry X. (2016). The cost-effectiveness of tumor-treating fields therapy in patients with newly diagnosed glioblastoma. Neuro Oncol..

[250] Zhu J.-J., Demireva P., Kanner A.A., Pannullo S., Mehdorn M., Avgeropoulos N., Salmaggi A., Silvani A., Goldlust S., David C. (2017). Health-related quality of life, cognitive screening, and functional status in a randomized phase III trial (EF-14) of tumor treating fields with temozolomide compared to temozolomide alone in newly diagnosed glioblastoma. J. Neuro Oncol..

[251] Connock M., Auguste P., Dussart C., Guyotat J., Armoiry X. (2019). Cost-effectiveness of tumor-treating fields added to maintenance temozolomide in patients with glioblastoma: An updated evaluation using a partitioned survival model. J. Neuro Oncol..

[252] Mooney J., Bernstock J.D., Ilyas A., Ibrahim A., Yamashita D., Markert J.M., Nakano I. (2019). Current Approaches and Challenges in the Molecular Therapeutic Targeting of Glioblastoma. World Neurosurg..

[253] Mu Q., Jeon M., Hsiao M.H., Patton V.K., Wang K., Press O.W., Zhang M. (2015). Stable and Efficient Paclitaxel Nanoparticles for Targeted Glioblastoma Therapy. Adv. Healthc. Mater..

[254] Mu Q., Lin G., Patton V.K., Wang K., Press O.W., Zhang M. (2015). Gemcitabine and chlorotoxin conjugated iron oxide nanoparticles for glioblastoma therapy. J. Mater. Chem..

[255] Hori Y.S., Hosoda R., Akiyama Y., Sebori R., Wanibuchi M., Mikami T., Sugino T., Suzuki K., Maruyama M., Tsukamoto M. (2015). Chloroquine potentiates temozolomide cytotoxicity by inhibiting mitochondrial autophagy in glioma cells. J. Neuro Oncol..

[256] Liu L.Q., Wang S.B., Shao Y.F., Shi J.N., Wang W., Chen W.Y., Ye Z.Q., Jiang J.Y., Fang Q.X., Zhang G.B. (2019). Hydroxychloroquine potentiates the anti-cancer effect of bevacizumab on glioblastoma via the inhibition of autophagy. Biomed. Pharmacother..

[257] Ye H., Chen M., Cao F., Huang H., Zhan R., Zheng X. (2016). Chloroquine, an autophagy inhibitor, potentiates the radiosensitivity of glioma initiating cells by inhibiting autophagy and activating apoptosis. BMC Neurol..

[258] Gonçalves R.M., Agnes J.P., Delgobo M., de Souza P.O., Thomé M.P., Heimfarth L., Lenz G., Moreira J.C.F., Zanotto-Filho A. (2019). Late autophagy inhibitor chloroquine improves efficacy of the histone deacetylase inhibitor SAHA and temozolomide in gliomas. Biochem. Pharmacol..

[259] Roy L.-O., Poirier M.-B., Fortin D. (2015). Chloroquine inhibits the malignant phenotype of glioblastoma partially by suppressing TGF-beta. Investig. New Drugs.

[260] Rosenfeld M.R., Ye X., Supko J.G., Desideri S., Grossman S.A., Brem S., Mikkelson T., Wang D., Chang Y.C., Hu J. (2014). A phase I/II trial of hydroxychloroquine in conjunction with radiation therapy and concurrent and adjuvant temozolomide in patients with newly diagnosed glioblastoma multiforme. Autophagy.

[261] Compter I., Eekers D.B., Hoeben A., Rouschop K.M., Reymen B., Ackermans L., Beckervordersantforth J., Bauer N.J., Anten M.M., Wesseling P. (2020). Chloroquine combined with concurrent radiotherapy and temozolomide for newly diagnosed glioblastoma: A phase IB trial. Autophagy.

[262] Shahcheraghi S.H., Tchokonte-Nana V., Lotfi M., Lotfi M., Ghorbani A., Sadeghnia H.R. (2020). Wnt/beta-catenin and PI3K/Akt/mtor Signaling Pathways in Glioblastoma: Two main targets for drug design: A Review. Curr. Pharm. Des..

[263] Seliger C., Luber C., Gerken M., Schaertl J., Proescholdt M., Riemenschneider M.J., Meier C.R., Bogdahn U., Leitzmann M.F., Klinkhammer-Schalke M. (2019). Use of metformin and survival of patients with high-grade glioma. Int. J. Cancer.

[264] Mazurek M., Litak J., Kamieniak P., Kulesza B., Jonak K., Baj J., Grochowski C. (2020). Metformin as Potential Therapy for High-Grade Glioma. Cancers.

[265] Kamarudin M.N., Parhar I. (2019). Emerging therapeutic potential of anti-psychotic drugs in the management of human glioma: A comprehensive review. Oncotarget.

[266] Tan S.K., Jermakowicz A., Mookhtiar A.K., Nemeroff C.B., Schürer S.C., Ayad N.G. (2018). Drug repositioning in glioblastoma: A pathway perspective. Front. Pharmacol..

[267] Rundle-Thiele D., Head R., Cosgrove L., Martin J.H. (2016). Repurposing some older drugs that cross the blood–brain barrier and have potential anticancer activity to provide new treatment options for glioblastoma. Br. J. Clin. Pharmacol..

[268] Adamski V., Schmitt C., Ceynowa F., Adelung R., Lucius R., Synowitz M., Hattermann K., Held-Feindt J. (2018). Effects of sequentially applied single and combined temozolomide, hydroxychloroquine and AT101 treatment in a long-term stimulation glioblastoma in vitro model. J. Cancer Res. Clin. Oncol..

[269] Boojar M. (2020). An Overview of the Cellular Mechanisms of Flavonoids Radioprotective Effects. Adv. Pharm. Bull..

[270] Hosseinzadeh E., Hassanzadeh A., Marofi F., Alivand M.R., Solali S. (2020). Flavonoid-Based Cancer Therapy: An Updated Review. Anti Cancer Agents Med. Chem..

[271] Chabot G.G., Touil Y.S., Pham M.H., Dauzonne D. (2010). Flavonoids in Cancer Prevention and Therapy: Chemistry, Pharmacology, Mechanisms of Action, and Perspectives for Cancer Drug Discovery. Alternative and Complementary Therapies for Cancer.

[272] Atiq A., Parhar I. (2020). Anti-neoplastic Potential of Flavonoids and Polysaccharide Phytochemicals in Glioblastoma. Molecules.

[273] Batra P., Sharma A. (2013). Anti-cancer potential of flavonoids: Recent trends and future perspectives. Biotech.

[274] Tavana E., Mollazadeh H., Mohtashami E., Modaresi S.M.S., Hosseini A., Sabri H., Soltani A., Javid H., Afshari A.R., Sahebkar A. (2020). Quercetin: A promising phytochemical for the treatment of glioblastoma multiforme. BioFactors.

[275] Fang D., Xiong Z., Xu J., Yin J., Luo R. (2019). Chemopreventive mechanisms of galangin against hepatocellular carcinoma: A review. Biomed. Pharmacother..

[276] Kong Y., Feng Z., Chen A., Qi Q., Han M., Wang S., Zhang Y. (2019). The Natural Flavonoid Galangin Elicits Apoptosis, Pyroptosis, and Autophagy in Glioblastoma. Front. Oncol..

[277] Chien S., Shi M.D., Lee Y., Te C., Shih Y. (2015). Galangin, a novel dietary flavonoid, attenuates metastatic feature via PKC/ERK signaling pathway in TPA-treated liver cancer HepG2 cells. Cancer Cell Int..

[278] Anand P., Kunnumakkara A.B., Newman R., Aggarwal B. (2007). Bioavailability of curcumin: Problems and promises. Mol. Pharm..

[279] Wang L.X., Ye X., Cai X., Su J., Ma R., Yin X., Zhou X.X., Li H., Wang Z. (2015). Curcumin suppresses cell growth and invasion and induces apoptosis by down-regulation of Skp2 pathway in glioma cellsE. Oncotarget.

[280] Fratantonio D., Molonia M.S., Bashllari R., Muscarà C., Ferlazzo G., Costa G., Saija A., Cimino F., Speciale A. (2019). Curcumin potentiates the antitumor activity of Paclitaxel in rat glioma C6 cells. Phytomedicine.

[281] Gersey Z.C., Rodriguez G.A., Barbarite E., Sanchez A., Walters W.M., Ohaeto K.C., Komotar R.J., Graham R.M. (2017). Curcumin decreases malignant characteristics of glioblastoma stem cells via induction of reactive oxygen species. BMC Cancer.

[282] Li B., Wang Y., Li S., He H., Sun F., Wang C., Lu Y., Wang X., Tao B. (2015). Decreased expression of miR-378 correlates with tumor invasiveness and poor prognosis of patients with glioma. Int. J. Clin. Exp. Pathol..

[283] Kutanzi K.R., Yurchenko O.V., Beland F.A., Checkhun V.F., Pogribny I.P. (2011). MicroRNA-mediated drug resistance in breast cancer. Clin. Epigenet..

[284] Ma J., Dong C., Ji C. (2010). MicroRNA and drug resistance. Cancer Gene Ther..

[285] Li W., Yang W., Liu Y., Chen S., Chin S.M., Qi X.L., Zhao Y., Liu H., Wang J., Mei X. (2017). MicroRNA-378 enhances inhibitory effect of curcumin on glioblastoma. Oncotarget.

[286] Tsai Y.-M., Chien C.-F., Lin L.-C., Tsai T.-H. (2011). Curcumin and its nano-formulation: The kinetics of tissue distribution and blood–brain barrier penetration. Int. J. Pharm..

[287] Mulik R.S., Mönkkönen J., Juvonen R.O., Mahadik K.R., Paradkar A.R. (2012). ApoE3 mediated polymeric nanoparticles containing curcumin: Apoptosis induced in vitro anticancer activity against neuroblastoma cells. Int. J. Pharm..

[288] Chen Y., Pan L., Jiang M., Li D., Jin L. (2016). Nanostructured lipid carriers enhance the bioavailability and brain cancer inhibitory efficacy of curcumin both in vitro and in vivo. Drug Deliv..

[289] Wu Q., Needs P.W., Lu Y., Kroon P.A., Ren D., Yang X. (2018). Different antitumor effects of quercetin, quercetin-3-sulfate and quercetin-3-glucuronide in human breast cancer MCF-7 cells. Food Funct..

[290] Khan F., Niaz K., Maqbool F., Hassan F., Abdollahi M., Venkata K., Nabavi S., Bishayee A. (2016). Molecular Targets Underlying the Anticancer Effects of Quercetin: An Update. Nutrients.

[291] Reyes-Farias M., Carrasco-Pozo C. (2019). The Anti-Cancer Effect of Quercetin: Molecular Implications in Cancer Metabolism. Int. J. Mol. Sci..

[292] Rauf A., Imran M., Khan I.A., Ur-Rehman M., Gilani S.A., Mehmood Z., Mubarak M.S. (2018). Anticancer potential of quercetin: A comprehensive review. Phytother. Res..

[293] Sang D.P., Li R.J., Lan Q. (2014). Quercetin sensitizes human glioblastoma cells to temozolomide in vitro via inhibition of Hsp27. Acta Pharmacol. Sin..

[294] Jang E., Kim I.Y., Kim H., Lee D.M., Seo D.Y., Lee J.A., Choi K.S., Kim E. (2020). Quercetin and chloroquine synergistically kill glioma cells by inducing organelle stress and disrupting Ca^2+^ homeostasis. Biochem. Pharmacol..

[295] Taylor M., Khathayer F., Ray S. (2019). Quercetin and Sodium Butyrate Synergistically Increase Apoptosis in Rat C6 and Human T98G Glioblastoma Cells Through Inhibition of Autophagy. Neurochem. Res..

[296] Wang G., Dai F., Yu K., Jia Z., Zhang A., Huang Q., Kang C., Jiang H., Pu P. (2015). Resveratrol inhibits glioma cell growth via targeting oncogenic microRNAs and multiple signaling pathways. Int. J. Oncol..

[297] Liu Y., Song X., Wu M., Wu J., Liu J. (2020). Synergistic Effects of Resveratrol and Temozolomide Against Glioblastoma Cells: Underlying Mechanism and Therapeutic Implications. Cancer Manag. Res..

[298] Richard S. (2019). The Therapeutic Potential of Resveratrol in Gliomas. Adv. Biosci. Clin. Med..

[299] Kiskova T., Kubatka P., Büsselberg D., Kassayova M. (2020). The Plant-Derived Compound Resveratrol in Brain Cancer: A Review. Biomolecules.

[300] Yuan Y., Xue X., Guo R.B., Sun X.L., Hu G. (2012). Resveratrol Enhances the Antitumor Effects of Temozolomide in Glioblastoma via ROS-dependent AMPK-TSC-mTOR Signaling Pathway. CNS Neurosci. Ther..

[301] Shu X.H., Wang L.L., Li H., Song X., Shi S., Gu J.Y., Wu M.L., Chen X.Y., Kong Q.Y., Liu J. (2015). Diffusion Efficiency and Bioavailability of Resveratrol Administered to Rat Brain by Different Routes: Therapeutic Implications. Neurotherapeutics.

[302] Song X., Shu X.-H., Wu M.-L., Zheng X., Jia B., Kong Q.-Y., Liu J., Li H. (2018). Postoperative resveratrol administration improves prognosis of rat orthotopic glioblastomas. BMC Cancer.

[303] Vijayakumar M.R., Vajanthri K.Y., Balavigneswaran C.K., Mahto S.K., Mishra N., Muthu M.S., Singh S. (2016). Pharmacokinetics, biodistribution, in vitro cytotoxicity and biocompatibility of Vitamin E TPGS coated trans resveratrol liposomes. Coll. Surf..

[304] Johnsen K., Burkhart A., Melander F., Kempen P., Vejlebo J., Siupka P., Nielsen M., Andresen T., Moos T. (2017). Targeting transferrin receptors at the blood-brain barrier improves the uptake of immunoliposomes and subsequent cargo transport into the brain parenchyma. Sci. Rep..

[305] Jhaveri A., Deshpande P., Pattni B., Torchilin V. (2018). Transferrin-targeted, resveratrol-loaded liposomes for the treatment of glioblastoma. J. Control. Release.

[306] Mukherjee S., Baidoo J.N.E., Sampat S., Mancuso A., David L., Cohen L.S., Zhou S., Banerjee P. (2018). Liposomal TriCurin, A Synergistic Combination of Curcumin, Epicatechin Gallate and Resveratrol, Repolarizes Tumor-Associated Microglia/Macrophages, and Eliminates Glioblastoma (GBM) and GBM Stem Cells. Molecules.

[307] Ravi R., Bedi A. (2004). NF-kappa B in cancer-a friend turned foe. Drug Resist. Update.

[308] Lemieszek M., Rzeski W. (2012). Anticancer properties of polysaccharides isolated from fungi of the Basidiomycetes class. Contemp. Oncol..

[309] Khan T., Date A., Chawda H., Patel K. (2019). Polysaccharides as potential anticancer agents—A review of their progress. Carbohydr. Polym..

[310] Zhou B., Fu Q., Song S.S., Zheng H.L., Wei Y.Z. (2015). Inhibitory effect of schizophyllan on rat glioma cells. Bangladesh J. Pharmacol..

[311] Luthuli S., Wu S., Cheng Y., Zheng X., Wu M., Tong H. (2019). Therapeutic Effects of Fucoidan: A Review on Recent Studies. Mar. Drugs.

[312] Yoo H.J., You D.J., Lee K.W. (2019). Characterization and Immunomodulatory Effects of High Molecular Weight Fucoidan Fraction from the Sporophyll of Undaria pinnatifida in Cyclophosphamide-Induced Immunosuppressed Mice. Mar. Drugs.

[313] Zhang J., Tang Q., Zhou C., Jia W., Da Silva L., Nguyen L.D., Reutter W., Fan H. (2010). GLIS, a bioactive proteoglycan fraction from Ganoderma lucidum, displays anti-tumour activity by increasing both humoral and cellular immune response. Life Sci..

[314] Wang C., Shi S., Chen Q., Lin S., Wang R., Wang S., Chen C. (2018). Antitumor and Immunomodulatory Activities of Ganoderma lucidum Polysaccharides in Glioma-Bearing Rats. Integr. Cancer Ther..

[315] Li A., Shuai X., Jia Z., Li H., Liang X., Su D., Guo W. (2015). Ganoderma lucidum polysaccharide extract inhibits hepatocellular carcinoma growth by downregulating regulatory T cells accumulation and function by inducing microRNA-125b. J. Transl. Med..

[316] Wang C., Lin D., Chen Q., Lin S., Shi S., Chen C. (2018). Polysaccharide peptide isolated from grass-cultured induces anti-proliferative and pro-apoptotic effects in the human U251 glioma cell line. Oncol. Lett..

[317] Wang K.-P., Zhang Q.-L., Liu Y., Wang J., Cheng Y., Zhang Y. (2013). Structure and Inducing Tumor Cell Apoptosis Activity of Polysaccharides Isolated from Lentinus edodes. J. Agric. Food Chem..

[318] Chen S.-N., Ching-Sheng C., Chen S., Wang W., Cheng-Jeng T., Chung-Lun L., Kim H. (2014). The Effect of Mushroom Beta-Glucans from Solid Culture of Ganoderma lucidum on Inhibition of the Primary Tumor Metastasis. Evid. Based Complement. Altern. Med..

[319] Huijeong A., Eunsaem J., Jin-Chul K., Seung Goo K., Sung-Il Y., Hyun-Jeong K., Pyeung-Hyeun K., Geun-Shik L. (2017). Lentinan from shiitake selectively attenuates AIM2 and non-canonical inflammasome activation while inducing pro-inflammatory cytokine production. Sci. Rep..

[320] Xu H.L., Dai J.H., Hu T., Liao Y.F. (2018). Lentinan up-regulates microRNA-340 to promote apoptosis and autophagy of human osteosarcoma cells. Int. J. Clin. Exp. Pathol..

[321] Xu H., Zou S., Xu X. (2017). The β-glucan from Lentinus edodes suppresses cell proliferation and promotes apoptosis in estrogen receptor positive breast cancers. Oncotarget.

[322] Liu X., Li M., Li W., Wang Q., Zhang H. (2019). Combined Effect of Lentinan and Cisplatin on Cytokines IL-6, TNF-alpha, and TGF-beta in Tumor Therapy. Int. J. Polym. Sci..

[323] Li X., Zhang M. (2014). In vitro inhibitory effects of lentinan on rat glioma cells. Biomed. Res..

[324] Mortimer T., Mabin T., Engelbrecht A. (2019). Cannabinoids: The lows and the highs of chemotherapy-induced nausea and vomiting. Future Oncol..

[325] Hermanson D., Marnett L. (2011). Cannabinoids, endocannabinoids, and cancer. Cancer Metastas. Rev..

[326] Gado F., Digiacomo M., Macchia M., Bertini S., Manera C. (2018). Traditional Uses of Cannabinoids and New Perspectives in the Treatment of Multiple Sclerosis. Medicines.

[327] Erices J.I., Torres Á., Niechi I., Bernales I., Quezada C. (2018). Current natural therapies in the treatment against glioblastoma. Phytother. Res..

[328] Dumitru C., Sandalcioglu I., Karsak M. (2018). Cannabinoids in Glioblastoma Therapy: New Applications for Old Drugs. Front. Mol. Neurosci..

[329] Scott K.A., Dalgleish A.G., Liu W.M. (2014). The combination of cannabidiol and Δ 9 -tetrahydrocannabinol enhances the anticancer effects of radiation in an orthotopic murine glioma model. Mol. Cancer Ther..

[330] Torres S., Lorente M., Rodríguez-Fornés F., Hernández-Tiedra S., Salazar M., García-Taboada E., Barcia J., Guzmán M., Velasco G. (2011). A combined preclinical therapy of cannabinoids and temozolomide against glioma. Mol. Cancer Ther..

[331] López-Valero I., Saiz-Ladera C., Torres S., Hernández-Tiedra S., García-Taboada E., Rodríguez-Fornés F., Barba M., Dávila D., Salvador-Tormo N., Guzmán M. (2018). Targeting Glioma Initiating Cells with A combined therapy of cannabinoids and temozolomide. Biochem. Pharmacol..

[332] Schultz S., Beyer M. (2017). GW Pharmaceuticals Achieves Positive Results in Phase 2 Proof of Concept Study in Glioma.

[333] Goyal S., Prajapati C.P., Gore P., Patil C.R., Mahajan U., Sharma C., Talla S., Ojha S. (2017). Therapeutic Potential and Pharmaceutical Development of Thymoquinone: A Multitargeted Molecule of Natural Origin. Front. Pharmacol..

[334] Pazhouhi M., Sariri R., Khazaei M., Moradi M., Khazaei M. (2018). Synergistic effect of temozolomide and thymoquinone on human glioblastoma multiforme cell line (U87MG). J. Cancer Res. Ther..

[335] Khan M.A., Tania M., Wei C., Mei Z., Fu S., Cheng J., Xu J., Fu J. (2015). Thymoquinone inhibits cancer metastasis by downregulating TWIST1 expression to reduce epithelial to mesenchymal transition. Oncotarget.

[336] Peng L., Liu A., Shen Y., Xu H.-Z., Yang S.-Z., Ying X.-Z., Liao W., Liu H.-X., Lin Z.-Q., Chen Q.-Y. (2013). Antitumor and anti-angiogenesis effects of thymoquinone on osteosarcoma through the NF-κB pathway. Oncol. Rep..

[337] Khan M.A., Tania M., Fu S., Fu J. (2017). Thymoquinone, as an anticancer molecule: From basic research to clinical investigation. Oncotarget.

[338] Racoma I.O., Meisen W.H., Wang Q.-E., Kaur B., Wani A.A. (2013). Thymoquinone Inhibits Autophagy and Induces Cathepsin-Mediated, Caspase-Independent Cell Death in Glioblastoma Cells. PLoS ONE.

[339] Khan H. (2014). Medicinal Plants in Light of History: Recognized Therapeutic Modality. J. Evid. Based Complement. Altern. Med..

[340] Yool A.J., Ramesh S. (2020). Molecular Targets for Combined Therapeutic Strategies to Limit Glioblastoma Cell Migration and Invasion. Front. Pharmacol..

[341] Rayan A., Raiyn J., Falah M. (2017). Nature is the best source of anticancer drugs: Indexing natural products for their anticancer bioactivity. PLoS ONE.

[342] Navya P., Kaphle A., Srinivas S., Bhargava S., Rotello V., Daima H. (2019). Current trends and challenges in cancer management and therapy using designer nanomaterials. Nano Converg..

[343] Min Y., Caster J.M., Eblan M.J., Wang A.Z. (2015). Clinical Translation of Nanomedicine. Chem. Rev..

[344] Michael J.S., Lee B.-S., Zhang M., Yu J.S. (2018). Nanotechnology for Treatment of Glioblastoma Multiforme. J. Transl. Intern. Med..

[345] Miranda A., Blanco-Prieto M.J., Sousa J., Pais A., Vitorino C. (2017). Breaching barriers in glioblastoma. Part II: Targeted drug delivery and lipid nanoparticles. Int. J. Pharm..

[346] Šamec N., Zottel A., Videtič Paska A., Jovčevska I. (2020). Nanomedicine and immunotherapy: A step further towards precision medicine for glioblastoma. Molecules.

[347] Toy R., Roy K. (2016). Engineering nanoparticles to overcome barriers to immunotherapy. Bioeng. Transl. Med..

[348] Ananda S., Nowak A.K., Cher L., Dowling A., Brown C., Simes J., Rosenthal M.A. (2011). Phase 2 trial of temozolomide and pegylated liposomal doxorubicin in the treatment of patients with glioblastoma multiforme following concurrent radiotherapy and chemotherapy. J. Clin. Neurosci..

[349] Nordling-David M.M., Yaffe R., Guez D., Meirow H., Last D., Grad E., Salomon S., Sharabi S., Levi-Kalisman Y., Golomb G. (2017). Liposomal temozolomide drug delivery using convection enhanced delivery. J. Control. Release.

[350] Guo P., Moses-Gardner A., Huang J., Smith E.R., Moses M.A. (2019). ITGA2 as a potential nanotherapeutic target for glioblastoma. Sci. Rep..

[351] Khaw A.K., Sameni S., Venkatesan S., Kalthur G., Hande M.P. (2015). Plumbagin alters telomere dynamics, induces DNA damage and cell death in human brain tumour cells. Mutat. Res. Genet. Toxicol. Environ. Mutagen..

[352] Yang L., Wang Y., Guo H., Guo M. (2015). Synergistic Anti-Cancer Effects of Icariin and Temozolomide in Glioblastoma. Cell Biochem. Biophys..

[353] Bai Z.-L., Tay V., Guo S.-Z., Ren J., Shu M.-G. (2018). Silibinin Induced Human Glioblastoma Cell Apoptosis Concomitant with Autophagy through Simultaneous Inhibition of mTOR and YAP. BioMed Res. Int..

[354] You Y., Wang R., Shao N., Zhi F., Yang Y. (2019). Luteolin suppresses tumor proliferation through inducing apoptosis and autophagy via MAPK activation in glioma. OncoTargets Ther..

[355] Chakrabarti M., Ray S. (2016). Anti-tumor activities of luteolin and silibinin in glioblastoma cells: Overexpression of miR-7–1-3p augmented luteolin and silibinin to inhibit autophagy and induce apoptosis in glioblastoma in vivo. Int. J. Program. Cell Death.

[356] Hong J.-M., Kim J.-H., Kim H., Lee W.J., Hwang Y.-I. (2019). SB365, Saponin D Induces Caspase-Independent Cell Death and Augments the Anticancer Effect of Temozolomide in Glioblastoma Multiforme Cells. Molecules.

[357] Cao L., Qu D., Wang H., Zhang S., Jia C., Shi Z., Wang Z., Zhang J., Ma J. (2016). Toosendanin exerts an anti-cancer effect in glioblastoma by inducing estrogen receptor β- and p53-mediated apoptosis. Int. J. Mol. Sci..

[358] Franco Y., Okubo M., Torre A., Paiva P., Rosa M., Silva V., Reis R., Imamura P., de Carvalho J., Longato G. (2019). Coronarin D Induces Apoptotic Cell Death and Cell Cycle Arrest in Human Glioblastoma Cell Line. Molecules.

[359] Chen W.-L., Barszczyk A., Turlova E., Deurloo M., Liu B., Yang B.B., Rutka J.T., Feng Z.-P., Sun H.-S. (2015). Inhibition of TRPM7 by carvacrol suppresses glioblastoma cell proliferation, migration and invasion. Oncotarget.

[360] Tezcan G., Tunca B., Bekar A., Yalcin M., Sahin S., Budak F., Cecener G., Egeli U., Demir C., Guvenc G. (2015). Ficus carica Latex Prevents Invasion Through Induction of Let-7d Expression in GBM Cell Lines. Cell. Mol. Neurobiol..

[361] Gu H., Feng J., Wang H., Qian Y., Yang L., Chen J., Jin F., Shi Y., Lu S., Liu Y. (2016). Celastrus orbiculatus extract inhibits the migration and invasion of human glioblastoma cells in vitro. BMC Complement. Altern. Med..

[362] Ma J.-W., Zhang Y., Ye J.-C., Li R., Wen Y.-L., Huang J.-X., Zhong X.-Y. (2017). Tetrandrine Exerts a Radiosensitization Effect on Human Glioma through Inhibiting Proliferation by Attenuating ERK Phosphorylation. Biomol. Ther..

[363] Lin K., Gao Z., Shang B., Sui S., Fu Q. (2015). Osthole suppresses the proliferation and accelerates the apoptosis of human glioma cells via the upregulation of microRNA-16 and downregulation of MMP-9. Mol. Med. Rep..

[364] Miao J., Jiang Y., Wang D., Zhou J., Fan C., Jiao F., Liu B., Zhang J., Wang Y., Zhang Q. (2015). Trichosanthin suppresses the proliferation of glioma cells by inhibiting LGR5 expression and the Wnt/β-catenin signaling pathway. Oncol. Rep..

[365] Chen Y., Gao Z., Wang B., Xu R. (2016). Towards precision medicine-based therapies for glioblastoma: Interrogating human disease genomics and mouse phenotypes. BMC Genom..

[366] Logun M., Zhao W., Mao L., Karumbaiah L. (2018). Microfluidics in malignant glioma research and precision medicine. Adv. Biosyst..

[367] Fan Y., Nguyen D.T., Akay Y., Xu F., Akay M. (2016). Engineering a brain cancer chip for high-throughput drug screening. Sci. Rep..

[368] Akay M., Hite J., Avci N.G., Fan Y., Akay Y., Lu G., Zhu J.-J. (2018). Drug screening of human GBM spheroids in brain cancer chip. Sci. Rep..

[369] Gallego-Perez D., Chang L., Shi J., Ma J., Kim S.-H., Zhao X., Malkoc V., Wang X., Minata M., Kwak K.J. (2016). On-chip clonal analysis of glioma-stem-cell motility and therapy resistance. Nano Lett..

[370] Tsai H.F. (2020). Glioma on Chips Analysis of Glioma Cell Guidance and Interaction in Microfluidic-Controlled Microenvironment Enabled by Machine Learning. Ph.D. Thesis.

[371] Bagó J.R., Alfonso-Pecchio A., Okolie O., Dumitru R., Rinkenbaugh A., Baldwin A.S., Miller C.R., Magness S.T., Hingtgen S.D. (2016). Therapeutically engineered induced neural stem cells are tumour-homing and inhibit progression of glioblastoma. Nat. Commun..

[372] Ramakrishna R., Pisapia D. (2015). Recent molecular advances in our understanding of glioma. Cureus.

[373] Karsy M., Guan J., Cohen A.L., Jensen R.L., Colman H. (2017). New molecular considerations for glioma: IDH, ATRX, BRAF, TERT, H3 K27M. Curr. Neurol. Neurosci. Rep..

[374] Khani P., Nasri F., Khani Chamani F., Saeidi F., Sadri Nahand J., Tabibkhooei A., Mirzaei H. (2019). Genetic and epigenetic contribution to astrocytic gliomas pathogenesis. J. Neurochem..

[375] Reifenberger G., Wirsching H.-G., Knobbe-Thomsen C.B., Weller M. (2017). Advances in the molecular genetics of gliomas—implications for classification and therapy. Nat. Rev. Clin. Oncol..

[376] Prados M.D., Byron S.A., Tran N.L., Phillips J.J., Molinaro A.M., Ligon K.L., Wen P.Y., Kuhn J.G., Mellinghoff I.K., De Groot J.F. (2015). Toward precision medicine in glioblastoma: The promise and the challenges. Neuro Oncol..

